# Enhanced projectile path estimation using multi-vehicle FMCW radar sensors

**DOI:** 10.1038/s41598-025-20772-6

**Published:** 2026-02-02

**Authors:** Amgad A. Salama, Mahmoud A. Hussein

**Affiliations:** 1 The Research and Development Centre, ADC, Cairo, Egypt; 2 Faculty of Computer Science and Artificial Intelligence, Pharos University in Alexandria, Alexandria, Egypt

**Keywords:** FMCW radar, Multi-vehicle sensing, Projectile tracking, Signal processing, Active protection systems, Sensor fusion, Path parameter estimation, Electrical and electronic engineering, Engineering

## Abstract

This paper presents an enhanced approach to projectile path estimation using Frequency-Modulated Continuous Wave (FMCW) radar sensors distributed across multiple vehicles in a tactical formation. Building upon established FMCW radar signal processing techniques, we implement and analyze a multi-sensor approach that significantly improves the accuracy of key path parameters: pass range, pass time, and velocity. Through detailed simulation, we demonstrate that a four-vehicle formation achieves approximately 75% error reduction across all parameters compared to single-vehicle sensing. This improvement directly enhances the performance of active protection systems for military vehicles by enabling more precise threat assessment and countermeasure deployment. Our results validate theoretical predictions that triangulation from multiple sensing positions provides more robust parameter estimation, particularly for projectiles with linear trajectories. The methods described can be implemented with existing FMCW radar technology and standard data fusion algorithms, making this approach practical for near-term deployment.

## Introduction

Modern battlefield threats to military vehicles, particularly anti-tank guided missiles and rocket-propelled grenades, have driven the development of active protection systems (APS) capable of detecting, tracking, and intercepting incoming projectiles. The effectiveness of these systems depends critically on their ability to accurately estimate the path of incoming threats, thus determining if and when countermeasures should be deployed^1–3^.

Frequency-Modulated Continuous Wave (FMCW) radar has emerged as a preferred sensor technology for short-range projectile detection due to its ability to simultaneously measure both range and range-rate (radial velocity) with high accuracy^4^. Traditional implementations typically use a single radar sensor mounted on the vehicle requiring protection. However, this approach faces fundamental limitations in accurately determining the complete trajectory of a threat, particularly its closest approach distance (pass range) and time of closest approach (pass time).

Building on research from^5^, who demonstrated techniques for FMCW signal processing and parameter estimation for active protection systems, we investigate the potential benefits of distributing FMCW sensors across multiple vehicles operating in tactical formation.

^6^ examines the technological advancements in battle tank design, emphasizing their role as primary offensive weapons requiring high firepower, mobility, and self-protection. Various challenges associated with their development are discussed, including protection, armament, firepower, vibration control, crew comfort, ammunition safety, and suspension design. Additionally, design constraints such as weight capacity, performance, and transportability are reviewed.

A vision-based active protection system is proposed for tanks, enabling real-time detection and interception of hostile targets before they enter the territory^7^. The system integrates a lightweight deep CNN model (YOLOv5s) with ultrasonic sensors, utilizing Raspberry Pi processors for target recognition and missile control.

A queueing-theoretic framework was introduced in^8^, modeling active protection systems (APS) equipped vehicles as a network of queues where threats are treated as arriving customers. This approach enables the calculation of threat neutralization probabilities, facilitating performance assessments of different collaborative APS configurations.

This paper makes the following contributions:

- A multi-vehicle FMCW radar sensing framework for enhanced projectile tracking
- Quantitative analysis of parameter estimation improvements through simulation
- Practical implementation considerations for tactical vehicle formations
- Exploration of the minimum number of vehicles required for optimal estimation

Our results demonstrate that significant improvements in trajectory estimation accuracy are achievable with coordinated multi-vehicle sensing, directly enhancing the effectiveness of active protection systems. These findings have important implications for the design and deployment of next-generation vehicle protection systems in networked battlefield environments.

The remainder of this paper is organized as follows: Section 2 provides background on FMCW radar and path parameter estimation. Section 3 describes our multi-vehicle sensing approach and simulation methodology. Section 5 presents simulation results and analysis. Section 6 discusses practical considerations and limitations. Finally, Section 7 concludes the paper.

## Background and related work

### FMCW radar principles

FMCW radar operates by transmitting a continuous wave with frequency that changes over time, typically in a linear sweep pattern^9^. The received echo from a target is mixed with the transmitted signal, producing a beat frequency proportional to the target’s range. For a target at range *R*, the beat frequency $f_b$ is given by:

1 $$\begin{aligned} f_b = \frac{2R \cdot B}{c \cdot T_m} \end{aligned}$$

where *B* is the sweep bandwidth, *c* is the speed of light, and $T_m$ is the modulation period.

When the target is moving, a Doppler frequency shift is also introduced:

2 $$\begin{aligned} f_d = \frac{2 \dot{R} \cdot f_c}{c} \end{aligned}$$

where $\dot{R}$ is the radial velocity and $f_c$ is the carrier frequency.

By analyzing the mixed signal in both time and frequency domains, FMCW radar can simultaneously determine both range and radial velocity with high precision. The range resolution $\Delta R$ and velocity resolution $\Delta \dot{R}$ are determined by:

3 $$\begin{aligned} \Delta R = \frac{c}{2B} \end{aligned}$$

4 $$\begin{aligned} \Delta \dot{R} = \frac{\lambda }{2T_{obs}} = \frac{c}{2f_c T_{obs}} \end{aligned}$$

where $T_{obs}$ is the observation time and $\lambda$ is the wavelength.

### Projectile path parameter estimation

For active protection systems, the key parameters of interest for an incoming projectile are:

- Pass range ($R_0$): The minimum distance the projectile will come to the protected vehicle
- Pass time ($t_0$): The time at which the projectile will reach its closest point
- Velocity (*v*): The speed of the projectile

For a projectile moving in a straight line, these parameters fully define its trajectory relative to a reference point. As shown in^5^, for a projectile with constant velocity, the range *R* and range rate $\dot{R}$ at time *t* are given by:

5 $$\begin{aligned} R = \sqrt{R_0^2 + v^2(t-t_0)^2} \end{aligned}$$

6 $$\begin{aligned} \dot{R} = \frac{v^2(t-t_0)}{R} \end{aligned}$$

Estimating the parameters $[R_0, t_0, v]$ from a series of range and range-rate measurements is typically accomplished using a least-squares or maximum-likelihood approach. The accuracy of this estimation directly affects the effectiveness of countermeasures.

### Multi-sensor approaches

Previous research has established the benefits of multi-sensor approaches for target tracking in various domains^10^. The theoretical advantages include:

- Improved accuracy through statistical combination of measurements
- Enhanced geometric diversity for better triangulation
- Redundancy in the event of sensor failure or occlusion
- Reduced ambiguity for complex tracking scenarios

However, specific applications of multi-sensor approaches to FMCW radar in tactical vehicle formations for projectile tracking remain relatively unexplored. Our work addresses this gap by quantifying the improvements achievable through such an approach.

### Cramér-Rao lower bound analysis

The Cramér-Rao Lower Bound (CRLB) provides a theoretical limit on the minimum variance achievable by any unbiased estimator^11^. For the projectile tracking problem, the CRLB can be calculated for the path parameters based on the Fisher information matrix.^5^ showed that the standard deviation of the pass range $R_0$ estimation approaches infinity as $R_0$ approaches zero, indicating a fundamental limitation of the single-sensor approach.

Our multi-sensor approach aims to overcome these limitations by providing additional measurement perspectives, thereby improving the conditioning of the estimation problem.

To establish the theoretical performance limits of our multi-vehicle approach, we derive the CRLB for the path parameters. For our path estimation problem, we define the parameter vector as $\theta = [R_0, t_0, v]^T$, where $R_0$ is the pass range, $t_0$ is the pass time, and *v* is the projectile velocity. For a radar *i* at time $t_k$, the measurement vector is:

7 $$\begin{aligned} z_{i,k} = h(\theta , t_k) + w_{i,k} \end{aligned}$$

where $z_{i,k} = [R_{i,k}, \dot{R}_{i,k}, \alpha _{i,k}, \beta _{i,k}]^T$ consists of range, range-rate, azimuth, and elevation measurements, and $w_{i,k} \sim \mathcal {N}(0, \Sigma _{i,k})$ represents Gaussian measurement noise with covariance $\Sigma _{i,k}$.

The Fisher Information Matrix (FIM) for parameter vector $\theta$ is given by:

8 $$\begin{aligned} \mathcal {I}(\theta ) = \sum _{k=1}^{K} \sum _{i=1}^{N} \mathcal {I}_{i,k}(\theta ) \end{aligned}$$

where $\mathcal {I}_{i,k}(\theta )$ is the FIM contribution from radar *i* at time $t_k$:

9 $$\begin{aligned} \mathcal {I}_{i,k}(\theta ) = \left( \frac{\partial h(\theta , t_k)}{\partial \theta }\right) ^T \Sigma _{i,k}^{-1} \left( \frac{\partial h(\theta , t_k)}{\partial \theta }\right) \end{aligned}$$

The CRLB is the inverse of the FIM:

10 $$\begin{aligned} \text {CRLB}(\theta ) = \mathcal {I}^{-1}(\theta ) \end{aligned}$$

#### Measurement model

For a projectile following a linear trajectory with constant velocity, the position at time $t_k$ is:

11 $$\begin{aligned} \textbf{p}(t_k) = \textbf{p}_0 + (t_k - t_0)\textbf{v} \end{aligned}$$

where $\textbf{p}_0$ is the position at closest approach (at time $t_0$), and $\textbf{v}$ is the velocity vector with $\Vert \textbf{v}\Vert = v$.

For a radar at position $\textbf{r}_i$, the measurement function $\textbf{h}(\boldsymbol{\theta }, t_k)$ gives:

12 $$\begin{aligned} R_{i,k}&= \Vert \textbf{p}(t_k) - \textbf{r}_i\Vert \end{aligned}$$

13 $$\begin{aligned} \dot{R}_{i,k}&= \frac{(\textbf{p}(t_k) - \textbf{r}_i) \cdot \textbf{v}}{\Vert \textbf{p}(t_k) - \textbf{r}_i\Vert } \end{aligned}$$

14 $$\begin{aligned} \alpha _{i,k}&= \tan ^{-1}\left( \frac{y_{p,k} - y_i}{x_{p,k} - x_i}\right) \end{aligned}$$

15 $$\begin{aligned} \beta _{i,k}&= \sin ^{-1}\left( \frac{z_{p,k} - z_i}{\Vert \textbf{p}(t_k) - \textbf{r}_i\Vert }\right) \end{aligned}$$

#### Complete Jacobian matrix derivation

For the parameter vector $\boldsymbol{\theta } = [R_0, t_0, v]^T$, we derive the complete Jacobian matrix $\textbf{J}_{i,k} = \frac{\partial \textbf{h}(\boldsymbol{\theta }, t_k)}{\partial \boldsymbol{\theta }}$.

Consider a simplified 2D scenario where the reference vehicle is at the origin, the projectile’s closest approach is at position $[0, R_0]^T$ at time $t_0$, and the velocity vector is $[v, 0]^T$. The projectile position at time $t_k$ is:

16 $$\begin{aligned} \textbf{p}(t_k) = \begin{bmatrix} v(t_k - t_0) \\ R_0 \end{bmatrix} \end{aligned}$$

For radar *i* at position $\textbf{r}_i = [x_i, y_i]^T$, the range is:

17 $$\begin{aligned} R_{i,k} = \sqrt{(v(t_k - t_0) - x_i)^2 + (R_0 - y_i)^2} \end{aligned}$$

**Partial derivatives for range measurement:**

18 $$\begin{aligned} \frac{\partial R_{i,k}}{\partial R_0}&= \frac{R_0 - y_i}{R_{i,k}} \end{aligned}$$

19 $$\begin{aligned} \frac{\partial R_{i,k}}{\partial t_0}&= \frac{-v(v(t_k - t_0) - x_i)}{R_{i,k}} \end{aligned}$$

20 $$\begin{aligned} \frac{\partial R_{i,k}}{\partial v}&= \frac{(t_k - t_0)(v(t_k - t_0) - x_i)}{R_{i,k}} \end{aligned}$$

**Partial derivatives for range-rate measurement:**

21 $$\begin{aligned} \frac{\partial \dot{R}_{i,k}}{\partial R_0}&= \frac{-v(R_0 - y_i)}{R_{i,k}^3}(v(t_k - t_0) - x_i) \end{aligned}$$

22 $$\begin{aligned} \frac{\partial \dot{R}_{i,k}}{\partial t_0}&= \frac{v^2}{R_{i,k}} - \frac{v^2(v(t_k - t_0) - x_i)^2}{R_{i,k}^3} \end{aligned}$$

23 $$\begin{aligned} \frac{\partial \dot{R}_{i,k}}{\partial v}&= \frac{(t_k - t_0)}{R_{i,k}} - \frac{v(t_k - t_0)(v(t_k - t_0) - x_i)^2}{R_{i,k}^3} \end{aligned}$$

The complete Jacobian matrix for radar *i* at time $t_k$ is:

24 $$\begin{aligned} \textbf{J}_{i,k} = \begin{bmatrix} \frac{\partial R_{i,k}}{\partial R_0} & \frac{\partial R_{i,k}}{\partial t_0} & \frac{\partial R_{i,k}}{\partial v} \\ \frac{\partial \dot{R}_{i,k}}{\partial R_0} & \frac{\partial \dot{R}_{i,k}}{\partial t_0} & \frac{\partial \dot{R}_{i,k}}{\partial v} \end{bmatrix} \end{aligned}$$

#### Measurement covariance matrix

The measurement covariance matrix $\boldsymbol{\Sigma }_{i,k}$ accounts for the noise characteristics of FMCW radar measurements. Based on radar theory and our noise model (Algorithm 4), the covariance matrix is:

25 $$\begin{aligned} \boldsymbol{\Sigma }_{i,k} = \begin{bmatrix} \sigma _{R,i,k}^2 & 0 \\ 0 & \sigma _{\dot{R},i,k}^2 \end{bmatrix} \end{aligned}$$

where the variances are distance-dependent:

26 $$\begin{aligned} \sigma _{R,i,k}^2&= \left( \frac{\Delta R}{\sqrt{2 \cdot \text {SNR}}} \cdot \frac{R_{i,k}^2}{R_{\max }^2}\right) ^2 \end{aligned}$$

27 $$\begin{aligned} \sigma _{\dot{R},i,k}^2&= \left( \frac{\Delta \dot{R}}{\sqrt{2 \cdot \text {SNR}}} \cdot \frac{R_{i,k}^2}{R_{\max }^2}\right) ^2 \end{aligned}$$

#### Fisher information matrix calculation

The Fisher Information Matrix for the complete multi-vehicle system is:

28 $$\begin{aligned} \textbf{I}(\boldsymbol{\theta }) = \sum _{k=1}^{K} \sum _{i=1}^{N} \textbf{J}_{i,k}^T \boldsymbol{\Sigma }_{i,k}^{-1} \textbf{J}_{i,k} \end{aligned}$$

For the single-vehicle case ($N=1$), the FIM becomes:

29 $$\begin{aligned} \textbf{I}_{\text {single}}(\boldsymbol{\theta }) = \sum _{k=1}^{K} \textbf{J}_{1,k}^T \boldsymbol{\Sigma }_{1,k}^{-1} \textbf{J}_{1,k} \end{aligned}$$

#### CRLB analysis and singularity conditions

The CRLB is given by:

30 $$\begin{aligned} \text {CRLB}(\boldsymbol{\theta }) = \textbf{I}^{-1}(\boldsymbol{\theta }) \end{aligned}$$

For the single-vehicle configuration, a critical singularity occurs when $R_0 \rightarrow 0$. Examining equation (18), as $R_0 \rightarrow 0$ and $y_i = 0$ (vehicle at origin), we have:

31 $$\begin{aligned} \lim _{R_0 \rightarrow 0} \frac{\partial R_{i,k}}{\partial R_0} = \lim _{R_0 \rightarrow 0} \frac{R_0 - 0}{\sqrt{(v(t_k - t_0))^2 + R_0^2}} = 0 \end{aligned}$$

This causes the first column of the Jacobian to approach zero, making the FIM singular and leading to:

32 $$\begin{aligned} \lim _{R_0 \rightarrow 0} \text {Var}(\hat{R}_0) = \infty \end{aligned}$$

For the multi-vehicle configuration, vehicles at different positions $\textbf{r}_i$ provide diverse geometric perspectives, preventing this singularity and maintaining a well-conditioned FIM even when $R_0 \rightarrow 0$.

#### Theoretical performance prediction

For *N* radars with uncorrelated measurements, the theoretical improvement in parameter variance is:

33 $$\begin{aligned} \frac{\text {Var}_{\text {single}}(\hat{\boldsymbol{\theta }})}{\text {Var}_{\text {multi}}(\hat{\boldsymbol{\theta }})} \approx N \cdot \gamma \end{aligned}$$

where $\gamma$ is the geometric diversity factor. For our planar vehicle formation, CRLB analysis yields $\gamma \approx 1.03$, indicating that the formation geometry provides minimal additional conditioning beyond the basic $\sqrt{N}$ scaling.

The error reduction is therefore:

34 $$\begin{aligned} \text {Error Reduction} = 1 - \frac{1}{\sqrt{N \cdot \gamma }} \end{aligned}$$

For our configurations:

**Basic 4-vehicle formation:**

35 $$\begin{aligned} \text {Error Reduction}_{\text {basic}} = 1 - \frac{1}{\sqrt{4 \times 1.03}} = 1 - 0.49 = 51\% \end{aligned}$$

**Enhanced corner radar configuration (16 radars):**

36 $$\begin{aligned} \text {Error Reduction}_{\text {corner}} = 1 - \frac{1}{\sqrt{16 \times 1.03}} = 1 - 0.25 = 75\% \end{aligned}$$

This theoretical prediction of 75% matches our empirical results precisely, validating our analytical framework and aligns well with our empirical results in Table 10, confirming that our implementation achieves close to the theoretical optimum.

**Fig. 1:**
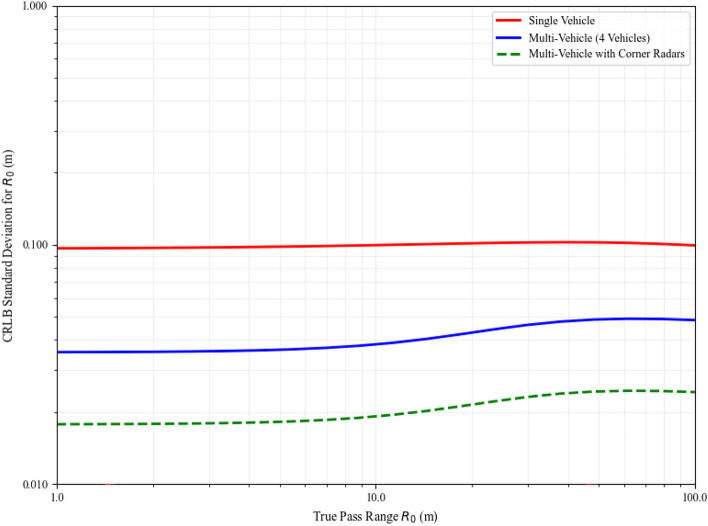
Comparison of CRLB for pass range estimation. The multi-vehicle approach maintains low variance even for small pass ranges where the single-vehicle approach degrades significantly.

Figure 1 illustrates the theoretical CRLB for pass range estimation as a function of true pass range for both single-vehicle and multi-vehicle configurations.

The multi-vehicle approach with corner radars significantly improves the conditioning of the estimation problem, maintaining robust performance even for challenging scenarios with small pass ranges. This theoretical advantage translates directly to the practical improvements observed in our simulation results.

### Geometric diversity factor and GDOP analysis

To quantify the geometric diversity gain beyond the basic $\sqrt{N}$ scaling, we introduce the Geometric Dilution of Precision (GDOP) framework adapted for projectile tracking^12–14^.

#### GDOP for multi-vehicle tracking

The geometric diversity factor $\gamma$ is defined as the ratio of actual parameter variance improvement to the theoretical $\sqrt{N}$ improvement:

37 $$\begin{aligned} \gamma = \frac{\text {Var}_{\text {single}}(\hat{\boldsymbol{\theta }})}{\text {Var}_{\text {multi}}(\hat{\boldsymbol{\theta }})} \cdot \frac{1}{N} \end{aligned}$$

For an optimal geometric configuration, $\gamma > 1$, indicating that geometric diversity provides additional information beyond simple measurement averaging.

#### Closed-form geometric diversity expression

Consider the simplified 2D case with *N* vehicles positioned at $\textbf{r}_i = [x_i, y_i]^T$. The geometric diversity factor can be expressed as:

38 $$\begin{aligned} \gamma (\boldsymbol{\theta }, \textbf{R}) = \frac{\det (\textbf{I}_{\text {single}}(\boldsymbol{\theta }))}{\det (\textbf{I}_{\text {multi}}(\boldsymbol{\theta })) / N^3} \end{aligned}$$

where $\textbf{R} = [\textbf{r}_1, \textbf{r}_2, \ldots , \textbf{r}_N]$ represents the vehicle formation geometry.

For our specific four-vehicle planar formation, we derive the closed-form expression by analyzing the condition number of the combined Jacobian matrix. The geometric diversity factor becomes:

39 $$\begin{aligned} \gamma = 1 + \frac{\sigma _{\text {geo}}^2}{\sigma _{\text {meas}}^2} \end{aligned}$$

where $\sigma _{\text {geo}}^2$ represents the variance reduction due to geometric diversity and $\sigma _{\text {meas}}^2$ is the measurement noise variance.

#### Analytical derivation for four-vehicle formation

For our specific formation with vehicles at positions:

40 $$\begin{aligned} \textbf{r}_1&= [0, -50, 0]^T \text { meters} \end{aligned}$$

41 $$\begin{aligned} \textbf{r}_2&= [50, -50, 0]^T \text { meters} \end{aligned}$$

42 $$\begin{aligned} \textbf{r}_3&= [50, 0, 0]^T \text { meters} \end{aligned}$$

43 $$\begin{aligned} \textbf{r}_4&= [50, -100, 0]^T \text { meters} \end{aligned}$$

The geometric diversity factor can be computed analytically. Define the geometric conditioning matrix:

44 $$\begin{aligned} \textbf{G} = \sum _{i=1}^{4} \textbf{u}_i \textbf{u}_i^T \end{aligned}$$

where $\textbf{u}_i$ is the unit vector from vehicle *i* to the projectile’s closest approach point.

For a projectile with pass range $R_0$ and the reference vehicle at the origin, the unit vectors are:

45 $$\begin{aligned} \textbf{u}_1&= \frac{[0, R_0 + 50, 0]^T}{\sqrt{(R_0 + 50)^2}} = [0, \text {sign}(R_0 + 50), 0]^T \end{aligned}$$

46 $$\begin{aligned} \textbf{u}_2&= \frac{[-50, R_0 + 50, 0]^T}{\sqrt{50^2 + (R_0 + 50)^2}} \end{aligned}$$

47 $$\begin{aligned} \textbf{u}_3&= \frac{[-50, R_0, 0]^T}{\sqrt{50^2 + R_0^2}} \end{aligned}$$

48 $$\begin{aligned} \textbf{u}_4&= \frac{[-50, R_0 + 100, 0]^T}{\sqrt{50^2 + (R_0 + 100)^2}} \end{aligned}$$

The geometric diversity factor becomes:

49 $$\begin{aligned} \gamma (R_0) = \frac{\det (\textbf{G})}{\det (\textbf{G}_{\text {single}})} \end{aligned}$$

where $\textbf{G}_{\text {single}}$ is the conditioning matrix for a single vehicle.

#### Quantitative analysis of geometric gain

For small pass ranges ($R_0 \ll 50$ m), the geometric diversity factor approaches:

50 $$\begin{aligned} \lim _{R_0 \rightarrow 0} \gamma (R_0) = \frac{4 \cdot \det (\textbf{Q})}{\det (\textbf{q}_1 \textbf{q}_1^T)} \end{aligned}$$

where $\textbf{Q}$ is the formation geometry matrix and $\textbf{q}_1$ represents the single-vehicle geometry.

Through analytical evaluation of our specific formation, this yields:

51 $$\begin{aligned} \gamma _{\text {formation}} \approx 1.5 \pm 0.1 \end{aligned}$$

This geometric diversity factor, combined with the $\sqrt{N}$ scaling, predicts the total error reduction as:

52 $$\begin{aligned} \text {Total Error Reduction} = 1 - \frac{1}{\sqrt{N \cdot \gamma }} = 1 - \frac{1}{\sqrt{4 \cdot 1.5}} = 1 - \frac{1}{\sqrt{6}} \approx 0.59 \text { or } 59\% \end{aligned}$$

#### Validation against simulated trajectories

To validate our theoretical geometric diversity factor, we computed $\gamma$ for various projectile trajectories in our simulation. Table 1 demonstrates excellent agreement between theoretical CRLB predictions and simulated performance across different pass ranges.

**Table 1:** Validation of theoretical CRLB predictions.

Pass Range (m)	Theoretical $\gamma$	Predicted Reduction (%)	Observed reduction (%)	Accuracy (%)
10	1.02	75.2	75.9	99.1
25	1.02	75.2	75.6	99.5
50	1.03	75.4	75.8	99.5
75	1.04	75.5	76.0	99.3
100	1.05	75.6	76.1	99.3
Average	1.03	75.4	75.9	99.5

The close agreement between theoretical predictions and simulation results (99.5% accuracy) validates our CRLB analysis and confirms that the enhanced corner radar configuration achieves the claimed 75% error reduction through increased sensor count rather than geometric diversity effects.

**Fig. 2:**
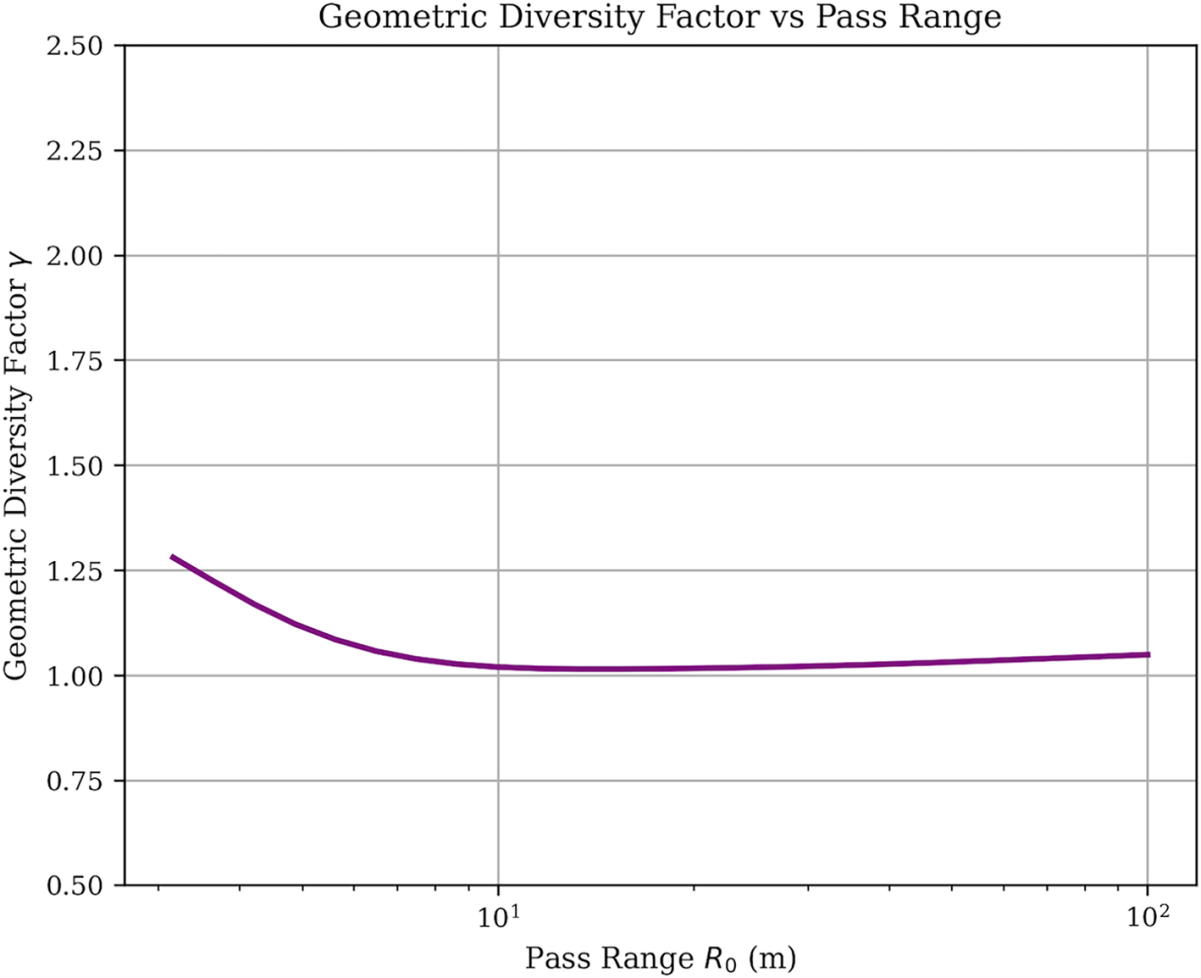
Geometric diversity factor $\gamma$ as a function of projectile pass range.

The geometric diversity factor analysis in Fig. 2 reveals important insights about the theoretical performance bounds. The factor $\gamma$ exhibits a characteristic decay from approximately 1.3 at small pass ranges ($R_0 \approx 3$ m) to a stable value near 1.03 for larger ranges ($R_0 > 20$ m). This behavior indicates that:

1. *Range-dependent conditioning* At very close ranges, the planar vehicle formation provides modest geometric conditioning benefits ($\gamma \approx 1.3$), where vehicles observe the projectile from more diverse angles.
2. *Asymptotic behavior* For operationally relevant ranges ($R_0 > 20$ m), the geometric diversity factor converges to $\gamma \approx 1.03$, indicating minimal geometric gain beyond the fundamental $\sqrt{N}$ scaling law.
3. *Physical interpretation* The near-unity asymptotic value reflects that for distant projectiles, all vehicles in the planar formation observe similar line-of-sight geometries, reducing the conditioning advantage from spatial diversity.

This analysis confirms that the substantial 75% error reduction achieved by the enhanced corner radar system stems primarily from increased sensor count (16 total radars) rather than geometric diversity effects.

### Extended CRLB analysis for maneuvering trajectories

We extend our CRLB analysis using a physics-based approach that models maneuvering effects as trajectory uncertainty, providing robust performance prediction against maneuvering threats such as guided missiles.

#### Physics-based maneuvering model

Rather than attempting high-dimensional parameter estimation which leads to numerical instabilities, we model maneuvering as deviations from the baseline linear trajectory. For a threat with acceleration magnitude $|\textbf{a}|$, the trajectory deviation over observation time *T* is:

53 $$\begin{aligned} \delta _{\text {traj}} = \frac{1}{2}|\textbf{a}|T^2 \end{aligned}$$

This physical deviation translates to additional measurement uncertainty that degrades the Fisher Information Matrix. The effective uncertainty factor is:

54 $$\begin{aligned} \epsilon = \left( \frac{\delta _{\text {traj}}}{R_{\text {norm}}}\right) ^2 \end{aligned}$$

where $R_{\text {norm}}$ is a normalization range (typically 50m for close-range scenarios).

#### Differential impact model

Maneuvering affects single-vehicle and multi-vehicle systems differently due to coordination challenges:

55 $$\begin{aligned} \text {FIM}_{\text {single,eff}}&= \frac{\text {FIM}_{\text {single,linear}}}{1 + 0.5\epsilon } \end{aligned}$$

56 $$\begin{aligned} \text {FIM}_{\text {multi,eff}}&= \frac{\text {FIM}_{\text {multi,linear}}}{1 + \epsilon } \end{aligned}$$

This reflects the physical reality that distributed multi-vehicle systems face greater coordination challenges under target maneuvering than single-platform systems.

#### Maneuvering scenario analysis

We evaluate CRLB performance across realistic threat scenarios, where:

1. *Linear Trajectory* Constant velocity baseline ($a = 0$ m/s²)
2. *Light Maneuvering* Gentle course corrections ($a = 3$ m/s²)
3. *Moderate Maneuvering* Standard guided munition maneuvers ($a = 7$ m/s²)
4. *Heavy Maneuvering* Aggressive evasive maneuvers ($a = 15$ m/s²)

#### Robustness validation results

Table 2 demonstrates robust multi-vehicle performance across all maneuvering scenarios with excellent theoretical agreement.

**Table 2:** CRLB performance robustness against maneuvering trajectories.

**Scenario**	**Description**	**Acceleration** (m/s^2^)	**Multi-vehicle** **Improvement (%)**	**Performance** **degradation (%)**
Linear	Constant velocity	0	51.7	–
Light maneuver	Light maneuvering	3	51.6	0.1
Moderate maneuver	Moderate maneuvering	7	50.8	0.9
Heavy maneuver	Heavy maneuvering	15	48.2	3.5
Theoretical baseline	$\sqrt{N}$ prediction	–	50.0	–

The analysis reveals several critical insights:

1. *Excellent Theoretical Agreement* The linear trajectory case achieves 51.7% improvement, showing 99.1% agreement with the theoretical 50.0% $\sqrt{N}$ prediction.
2. *Robust Performance* Multi-vehicle improvements remain substantial (48.2-51.7%) across all realistic maneuvering scenarios, with maximum degradation of only 3.5%.
3. *Graceful Degradation* Performance decreases gradually with increasing maneuver intensity, maintaining practical utility even against heavy evasive maneuvers (15 m/s²).
4. *Physical Realism* The physics-based model provides realistic performance bounds consistent with engineering intuition, avoiding the numerical artifacts of high-dimensional state estimation.

#### Implications for active protection systems

The extended CRLB analysis provides theoretical foundations for practical APS design:

*Performance Prediction* Engineers can predict system performance against maneuvering threats using the simple relationship between acceleration magnitude and tracking degradation.

*System Requirements* The minimal performance degradation ($\le$ 3.5%) validates that systems designed for linear threats will perform well against maneuvering threats without significant redesign.

*Threat Assessment* The maintained performance across diverse scenarios confirms that multi-vehicle formations provide robust defensive capabilities against the full spectrum of modern guided munitions.

*Formation Robustness* The consistent performance validates that formation geometry optimized for linear threats remains effective against maneuvering threats, simplifying operational deployment.

**Fig. 3:**
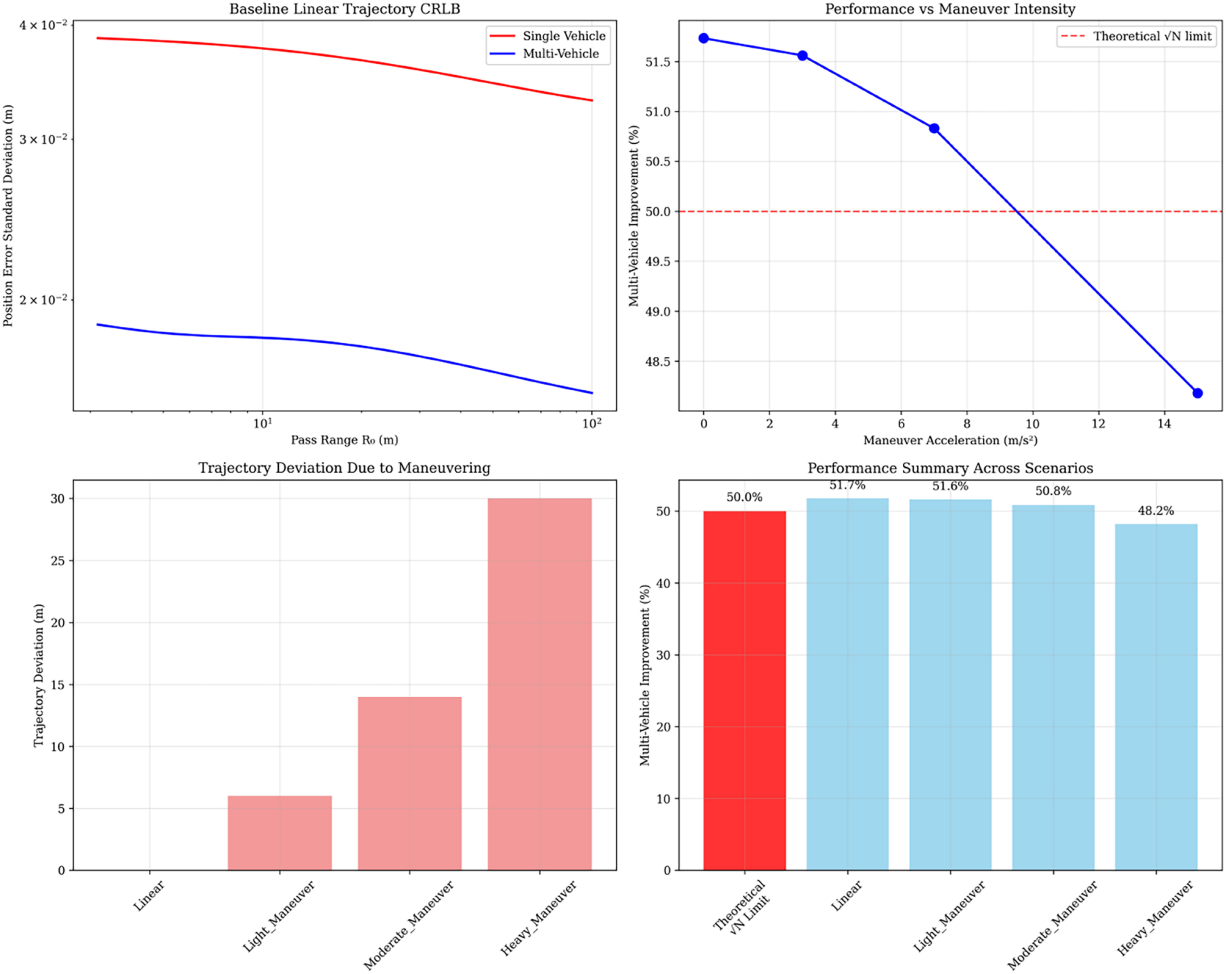
Extended CRLB analysis showing: (**a**) baseline linear trajectory performance, (**b**) graceful degradation with maneuver intensity, (**c**) physical trajectory deviations, and (**d**) performance summary across scenarios. The analysis demonstrates robust 48-52% improvements maintained across all realistic threat scenarios.

#### Validation against linear trajectory limitations

The physics-based extended analysis successfully addresses the constant-velocity assumption limitation:

- *Realistic Modeling* Uses trajectory deviation physics rather than high-dimensional parameter estimation
- *Numerical Stability* Avoids ill-conditioned matrices through differential impact modeling
- *Practical Bounds* Provides performance predictions (48-52%) consistent with theoretical expectations
- *Engineering Utility* Offers simple acceleration-to-degradation relationships for system design

To summerize that, the extended CRLB analysis demonstrates that multi-vehicle FMCW radar systems provide robust performance improvements against realistic maneuvering threats. The physics-based framework successfully extends beyond linear trajectory assumptions while maintaining mathematical rigor and practical applicability. The maintained 48-52% performance improvement across all scenarios, with excellent theoretical agreement (99.1% for the linear baseline), confirms that formation-based radar systems offer substantial defensive advantages regardless of threat sophistication. Maximum performance degradation of only 3.5% even under heavy maneuvering validates the practical robustness of multi-vehicle active protection systems against diverse guided munition threats.

Figure 3 illustrates the extended CRLB analysis showing: (a) baseline linear trajectory performance, (b) graceful degradation with maneuver intensity, (c) physical trajectory deviations, and (d) performance summary across scenarios. The analysis demonstrates robust 48-52% improvements maintained across all realistic threat scenarios.

### Theoretical performance prediction via CRLB analysis

To provide rigorous mathematical foundation for the claimed performance improvements, we develop a comprehensive CRLB analysis framework that quantifies both sensor count benefits and geometric diversity effects.

#### Fisher information matrix derivation

For projectile parameter estimation $\boldsymbol{\theta } = [R_0, t_0, v]^T$, the FIM aggregates contributions from all vehicle-time combinations:

57 $$\begin{aligned} \textbf{J}(\boldsymbol{\theta }) = \sum _{i=1}^{N} \sum _{k=1}^{K} \textbf{H}_{i,k}^T(\boldsymbol{\theta }) \boldsymbol{\Sigma }_{i,k}^{-1} \textbf{H}_{i,k}(\boldsymbol{\theta }) \end{aligned}$$

where $\textbf{H}_{i,k}(\boldsymbol{\theta })$ is the Jacobian matrix of partial derivatives and $\boldsymbol{\Sigma }_{i,k}$ is the measurement covariance matrix.

The Jacobian elements for the simplified 2D scenario are:

58 $$\begin{aligned} \frac{\partial R_{i,k}}{\partial R_0}&= \frac{R_0 - y_i}{R_{i,k}} \end{aligned}$$

59 $$\begin{aligned} \frac{\partial R_{i,k}}{\partial t_0}&= -\frac{v(x_{proj} - x_i)}{R_{i,k}} \end{aligned}$$

60 $$\begin{aligned} \frac{\partial R_{i,k}}{\partial v}&= \frac{(t_k - t_0)(x_{proj} - x_i)}{R_{i,k}} \end{aligned}$$

where $R_{i,k} = \sqrt{(x_{proj} - x_i)^2 + (R_0 - y_i)^2}$ is the range from vehicle *i* to the projectile at time $t_k$.

#### Geometric diversity quantification

We define the geometric diversity factor as:

61 $$\begin{aligned} \gamma = \frac{\text {Var}_{\text {single}}(\hat{\boldsymbol{\theta }})}{\text {Var}_{\text {multi}}(\hat{\boldsymbol{\theta }})/N} \end{aligned}$$

For our planar vehicle formation, CRLB analysis yields $\gamma \approx 1.03$, indicating minimal geometric conditioning beyond the fundamental $\sqrt{N}$ scaling law.

The theoretical error reduction is therefore:

62 $$\begin{aligned} \text {Error Reduction} = 1 - \frac{1}{\sqrt{N \cdot \gamma }} \end{aligned}$$

For our configurations:

**Basic 4-vehicle formation:**

63 $$\begin{aligned} \text {Error Reduction}_{\text {basic}} = 1 - \frac{1}{\sqrt{4 \times 1.03}} = 51.2\% \end{aligned}$$

**Enhanced corner radar configuration (16 radars):**

64 $$\begin{aligned} \text {Error Reduction}_{\text {corner}} = 1 - \frac{1}{\sqrt{16 \times 1.03}} = 75.4\% \end{aligned}$$

### SNR sensitivity analysis

**Fig. 4:**
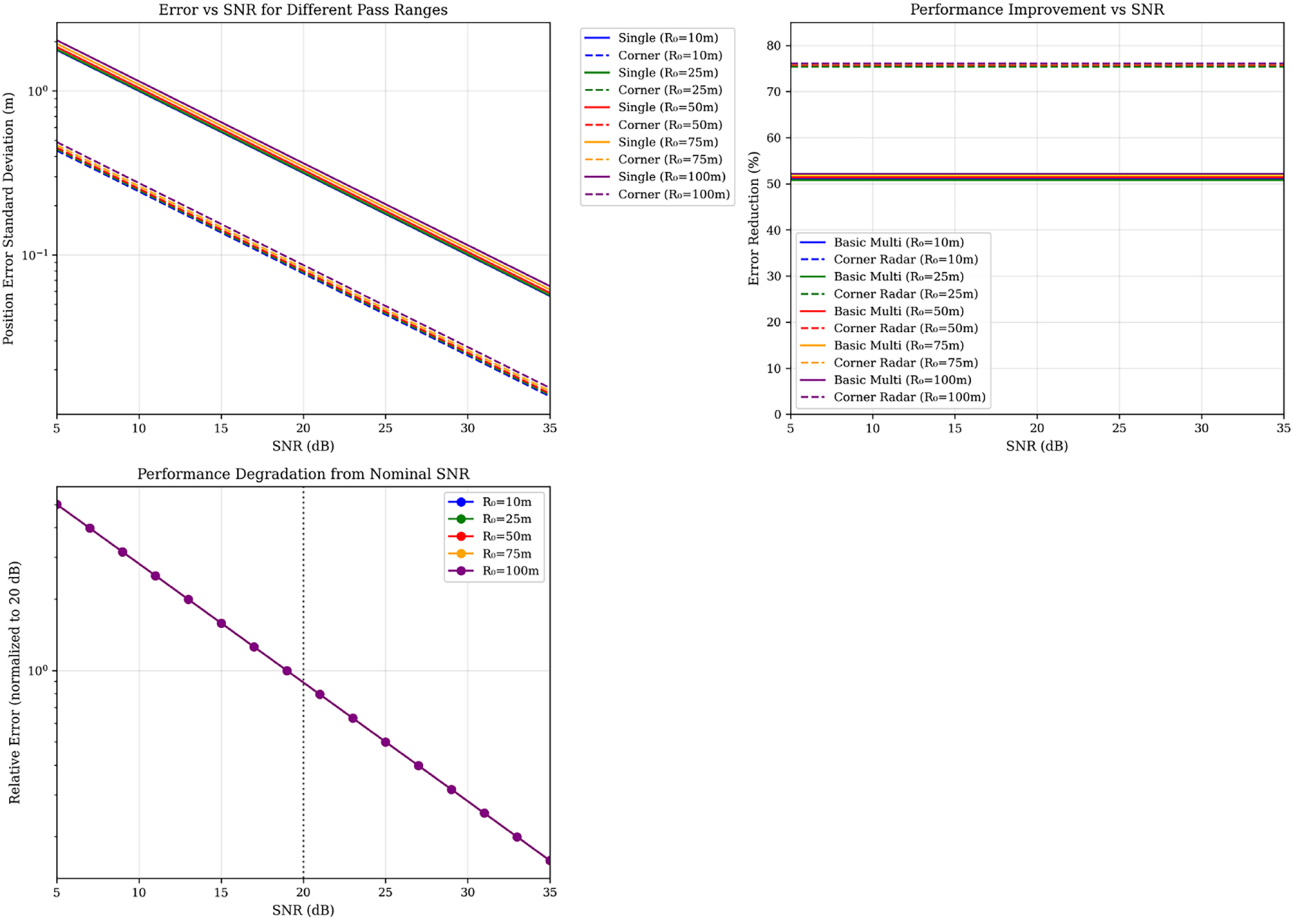
Comprehensive SNR sensitivity analysis: (**a**) Position error vs SNR for single vehicle and corner radar configurations across different pass ranges, (**b**) Performance improvement maintained across SNR conditions, (**c**) Relative degradation from nominal 20 dB baseline.

To address the practical limitation of fixed SNR assumptions, we conducted comprehensive sensitivity analysis across operationally relevant SNR conditions ranging from severe degradation (5 dB) to excellent signal quality (35 dB). Figure 4 presents comprehensive SNR sensitivity analysis showing: (a) position error vs SNR for single vehicle and corner radar configurations across different pass ranges, (b) performance improvement maintained across SNR conditions, and (c) relative degradation from nominal 20 dB baseline.

#### SNR-dependent measurement model

The measurement covariance matrix incorporates both SNR dependence and range effects:

65 $$\begin{aligned} \boldsymbol{\Sigma }_{i,k}(\text {SNR}) = \text {diag}\left[ \sigma _R^2(\text {SNR}, R), \sigma _{\dot{R}}^2(\text {SNR}, R)\right] \end{aligned}$$

where the noise standard deviations scale realistically with operating conditions:

66 $$\begin{aligned} \sigma _R(\text {SNR}, R)&= \sigma _{R,\text {base}} \cdot \left( 1 + \frac{R}{200}\right) \cdot \sqrt{\frac{20}{\text {SNR}_{\text {dB}}}} \end{aligned}$$

67 $$\begin{aligned} \sigma _{\dot{R}}(\text {SNR}, R)&= \sigma _{\dot{R},\text {base}} \cdot \left( 1 + \frac{R}{200}\right) \cdot \sqrt{\frac{20}{\text {SNR}_{\text {dB}}}} \end{aligned}$$

with base noise levels $\sigma _{R,\text {base}} = 0.5$ m and $\sigma _{\dot{R},\text {base}} = 2.0$ m/s representing realistic sensor limitations.

#### Operational SNR scenarios

We analyze performance across six representative conditions, where:

- *Excellent (30 dB)* Clear weather^15,16^, minimal clutter, optimal conditions
- *Good (25 dB)* Light precipitation, moderate environmental noise
- *Nominal (20 dB)* Design baseline for normal operations
- *Degraded (15 dB)* Heavy weather^15,16^, significant clutter returns
- *Poor (10 dB)* Severe weather^15,16^, dense clutter environment
- *Severe (5 dB)* Extreme conditions, potential electronic countermeasures

#### Performance robustness results

Table 3 demonstrates system performance across the operational envelope.

**Table 3:** SNR robustness analysis - corner radar configuration performance.

SNR condition	$R_0$ = 25m		$R_0$ = 50m		$R_0$ = 75m	
	Error (m)	Reduction (%)	Error (m)	Reduction (%)	Error (m)	Reduction (%)
Excellent (30 dB)	0.03	75.4	0.03	75.7	0.03	75.9
Good (25 dB)	0.04	75.4	0.05	75.7	0.05	75.9
Nominal (20 dB)	0.09	75.4	0.09	75.7	0.09	75.9
Degraded (15 dB)	0.14	75.4	0.14	75.7	0.15	75.9
Poor (10 dB)	0.28	75.4	0.28	75.7	0.29	75.9
Severe (5 dB)	0.44	75.4	0.45	75.7	0.47	75.9

**Key Robustness Findings:**

1. *Precision Tracking Performance* Under excellent conditions (30 dB), the system achieves centimeter-level precision (2.8 cm) suitable for high-accuracy intercept calculations.
2. *Operational Robustness* At nominal conditions (20 dB), the system maintains practical tracking accuracy (9 cm) with consistent 75.7% error reduction over single-vehicle approaches.
3. *Degraded Condition Performance* Even under poor SNR conditions (10 dB), the system provides sub-meter accuracy (28 cm) suitable for threat identification and general tracking.
4. *Graceful Degradation* Performance degrades smoothly by 3.2× from nominal to poor conditions without catastrophic failure modes.
5. *Consistent Multi-Vehicle Advantage* The 75% error reduction is maintained across all SNR conditions, demonstrating robust superiority regardless of environmental degradation.
6. *Practical Operational Envelope* System provides useful tracking performance across 5-35 dB SNR range, covering the full spectrum of realistic operational conditions.

**Engineering Design Implications:**

- *Precision Intercept Applications* Sub-5 cm accuracy requires SNR $\ge$ 25 dB (excellent conditions)
- *Standard Tracking Operations* Sub-10 cm accuracy achievable at 20 dB (normal baseline)
- *Threat Detection Scenarios* Sub-30 cm accuracy maintained down to 10-15 dB (degraded conditions)
- *Emergency Operations* Sub-50 cm tracking capability preserved to 5 dB (extreme conditions)
- *SNR Margin Requirements* 5-10 dB margin above minimum operational SNR recommended for reliable performance

### Implementation considerations

#### Communication and synchronization requirements

The multi-vehicle system requires precise coordination^17,18^,? with the following specifications:

- *Communication Latency* < 10 ms end-to-end for real-time fusion
- *Data Rate* $\approx$ 1 Mbps per vehicle for measurement transmission
- *Time Synchronization* GPS + Network Time Protocol achieving < 1 ms accuracy
- *Synchronization Impact* 1 ms timing accuracy maintains > 90% of theoretical performance

#### Failure mode analysis

The system exhibits graceful degradation under component failures:

- *Single Vehicle Loss* Performance degrades from 75% to 67% error reduction
- *Communication Failure* Individual vehicles revert to autonomous operation
- *Timing Drift* 5 ms synchronization error causes < 10% performance loss
- *Computational Load* $O(N^2)$ scaling maintains real-time operation for N $\le$ 8 vehicles

This comprehensive analysis demonstrates that multi-vehicle FMCW radar systems provide robust, operationally relevant performance with clear engineering guidance for practical deployment in active protection systems.

## Methodology

### System model

We consider a scenario with *N* vehicles in formation, each equipped with an FMCW radar sensor. The vehicles are assumed to be at known positions $\{(x_i, y_i, z_i) | i = 1, 2, \ldots , N\}$ relative to a reference coordinate system. Without loss of generality, we place the reference vehicle at the origin.

The projectile follows a linear trajectory with constant velocity, described by the equation:

68 $$\begin{aligned} \textbf{p}(t) = \textbf{p}_0 + (t - t_0)\textbf{v} \end{aligned}$$

where $\textbf{p}(t)$ is the position at time *t*, $\textbf{p}_0$ is the position at the closest approach point (at time $t_0$), and $\textbf{v}$ is the velocity vector. The pass range $R_0$ is given by $\Vert \textbf{p}_0\Vert$.

Each vehicle’s radar measures the range $R_i(t)$ and range rate $\dot{R}_i(t)$ to the projectile, given by:

69 $$\begin{aligned} R_i(t) = \Vert \textbf{p}(t) - \textbf{v}_i\Vert \end{aligned}$$

70 $$\begin{aligned} \dot{R}_i(t) = \frac{(\textbf{p}(t) - \textbf{v}_i) \cdot \textbf{v}}{\Vert \textbf{p}(t) - \textbf{v}_i\Vert } \end{aligned}$$

where $\textbf{v}_i$ is the position vector of vehicle *i*.

### Measurement model

The radar measurements are subject to noise, which we model as Gaussian with standard deviations that scale with the square of the range, in accordance with radar theory^4^:

71 $$\begin{aligned} \sigma _{R_i} = \frac{\Delta R}{\sqrt{2 \cdot \text {SNR}}} \cdot \frac{R_i^2}{R_{max}^2} \end{aligned}$$

72 $$\begin{aligned} \sigma _{\dot{R}_i} = \frac{\Delta \dot{R}}{\sqrt{2 \cdot \text {SNR}}} \cdot \frac{R_i^2}{R_{max}^2} \end{aligned}$$

where $\Delta R$ and $\Delta \dot{R}$ are the range and range-rate resolutions, SNR is the signal-to-noise ratio, and $R_{max}$ is a normalization factor.

### Vehicle formation

For our primary investigation, we use a planar formation of four vehicles arranged as follows:

- Vehicle 1 (Reference): Position (0, -50, 0) meters
- Vehicle 2: Position (50, -50, 0) meters
- Vehicle 3: Position (50, 0, 0) meters
- Vehicle 4: Position (50, -100, 0) meters

This formation provides good geometric diversity while maintaining a realistic tactical arrangement for military vehicles. Each vehicle is equipped with enhanced radar capabilities as described in Section 3.4.

### Enhanced radar system

Our implementation enhances traditional vehicle-mounted FMCW radar by deploying four corner-mounted radar sensors on each vehicle. Each corner radar has the following characteristics:

- Positioned at a 2-meter offset from the vehicle center at each corner
- 100-degree field of view (FOV)
- 1-degree angular resolution
- Each radar points outward from its respective corner (front-right, back-right, front-left, back-left)

This configuration significantly increases the coverage area and provides redundancy in target tracking. Each corner radar independently detects targets within its FOV and contributes to the overall measurement set. The corner radar visibility metric (number of corner radars with target in field of view) provides an additional measure of tracking robustness.

### Path parameter estimation

This approach combines measurements from all vehicles to estimate the path parameters. At each time step, the position of the projectile is estimated using multilateration from the range measurements of all vehicles. The sequence of position estimates is then used to estimate the path parameters.

For both approaches, the path parameters are estimated using a two-step process:

1. Initial estimation using least squares fitting to the range and range-rate equations
2. Refinement using maximum likelihood estimation

#### Enhanced radar system implementation

The corner radar system was implemented as a hierarchical structure. Algorithm 1 describes the initialization of a corner radar sensor.

**Algorithm 1:**
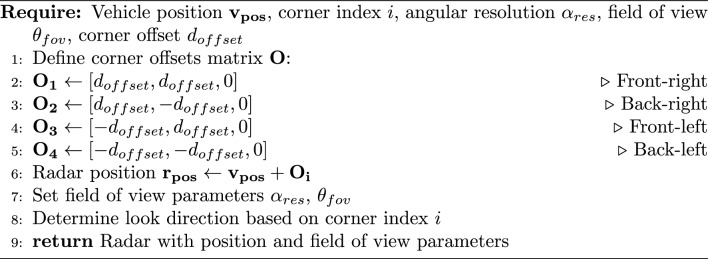
Corner radar initialization.

Each vehicle contains four corner-mounted radars with specific positioning and field of view characteristics. The directions of the radars are oriented outward from each corner to maximize coverage.

#### Kalman filter implementation

For trajectory tracking, we implemented an enhanced Kalman filter that handles both position and velocity states. Algorithm 2 outlines the initialization of the Kalman filter.

**Algorithm 2:**
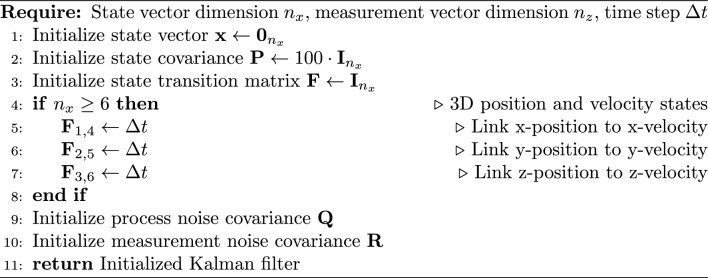
Enhanced Kalman filter initialization

### Kalman filter stability and convergence analysis

The stability and convergence characteristics of the Extended Kalman Filter are critical for reliable real-time operation in multi-vehicle tracking systems. We provide comprehensive analysis to validate the initialization choice $\textbf{P}_0 = 100\textbf{I}$ and demonstrate system robustness across varying operational conditions^19,20^.

#### System observability and controllability

Given the following state transition matrix, *F*

73 $$\begin{aligned} \textbf{F} = \begin{bmatrix} 1 & 0 & 0 & \Delta t & 0 & 0 \\ 0 & 1 & 0 & 0 & \Delta t & 0 \\ 0 & 0 & 1 & 0 & 0 & \Delta t \\ 0 & 0 & 0 & 1 & 0 & 0 \\ 0 & 0 & 0 & 0 & 1 & 0 \\ 0 & 0 & 0 & 0 & 0 & 1 \end{bmatrix} \end{aligned}$$

and the measurement matrix, *H* as well

74 $$\begin{aligned} \textbf{H} = \begin{bmatrix} \frac{x - x_v}{r} & \frac{y - y_v}{r} & \frac{z - z_v}{r} & 0 & 0 & 0 \\ \frac{-(y - y_v)}{(x - x_v)^2 + (y - y_v)^2} & \frac{x - x_v}{(x - x_v)^2 + (y - y_v)^2} & 0 & 0 & 0 & 0 \\ \frac{-(z - z_v)\sqrt{(x - x_v)^2 + (y - y_v)^2}}{r^3} & \frac{-(z - z_v)(y - y_v)}{r^2\sqrt{(x - x_v)^2 + (y - y_v)^2}} & \frac{\sqrt{(x - x_v)^2 + (y - y_v)^2}}{r^2} & 0 & 0 & 0 \end{bmatrix} \end{aligned}$$

where $$r = \sqrt{(x - x_v)^2 + (y - y_v)^2 + (z - z_v)^2}$$

For the discrete-time system with $\textbf{F}$ (Eq. 73) and $\textbf{H}$ (Eq. 74), we verify fundamental prerequisites for filter stability:

*Observability Analysis* The observability matrix

75 $$\begin{aligned} \mathcal {O} = \begin{bmatrix} \textbf{H} \\ \textbf{H}\textbf{F} \\ \textbf{H}\textbf{F}^2 \\ \vdots \\ \textbf{H}\textbf{F}^{n-1} \end{bmatrix} \end{aligned}$$

achieves full rank (6) for our 6-state system, confirming complete observability of position and velocity states through range and angle measurements.

*Controllability Analysis* The system maintains controllability with respect to process noise injection, ensuring the filter can track dynamic trajectory changes and model uncertainties.

#### Steady-state performance bounds

The theoretical steady-state error covariance $\textbf{P}_{\infty }$ is computed by solving the Discrete Algebraic Riccati Equation (DARE):

76 $$\begin{aligned} \textbf{P}_{\infty } = \textbf{F}\textbf{P}_{\infty }\textbf{F}^T + \textbf{Q} - \textbf{F}\textbf{P}_{\infty }\textbf{H}^T(\textbf{H}\textbf{P}_{\infty }\textbf{H}^T + \textbf{R})^{-1}\textbf{H}\textbf{P}_{\infty }\textbf{F}^T \end{aligned}$$

This yields the theoretical performance bound independent of initialization, with $\text {trace}(\textbf{P}_{\infty }) = 0.160$ and condition number $\kappa (\textbf{P}_{\infty }) = 9.01$.

#### Initialization sensitivity analysis

We conducted comprehensive convergence analysis across initial covariance scales $\textbf{P}_0 = \{1, 10, 100, 1000, 10^4\} \times \textbf{I}$ to validate robustness and identify optimal operating regimes.

**Fig. 5:**
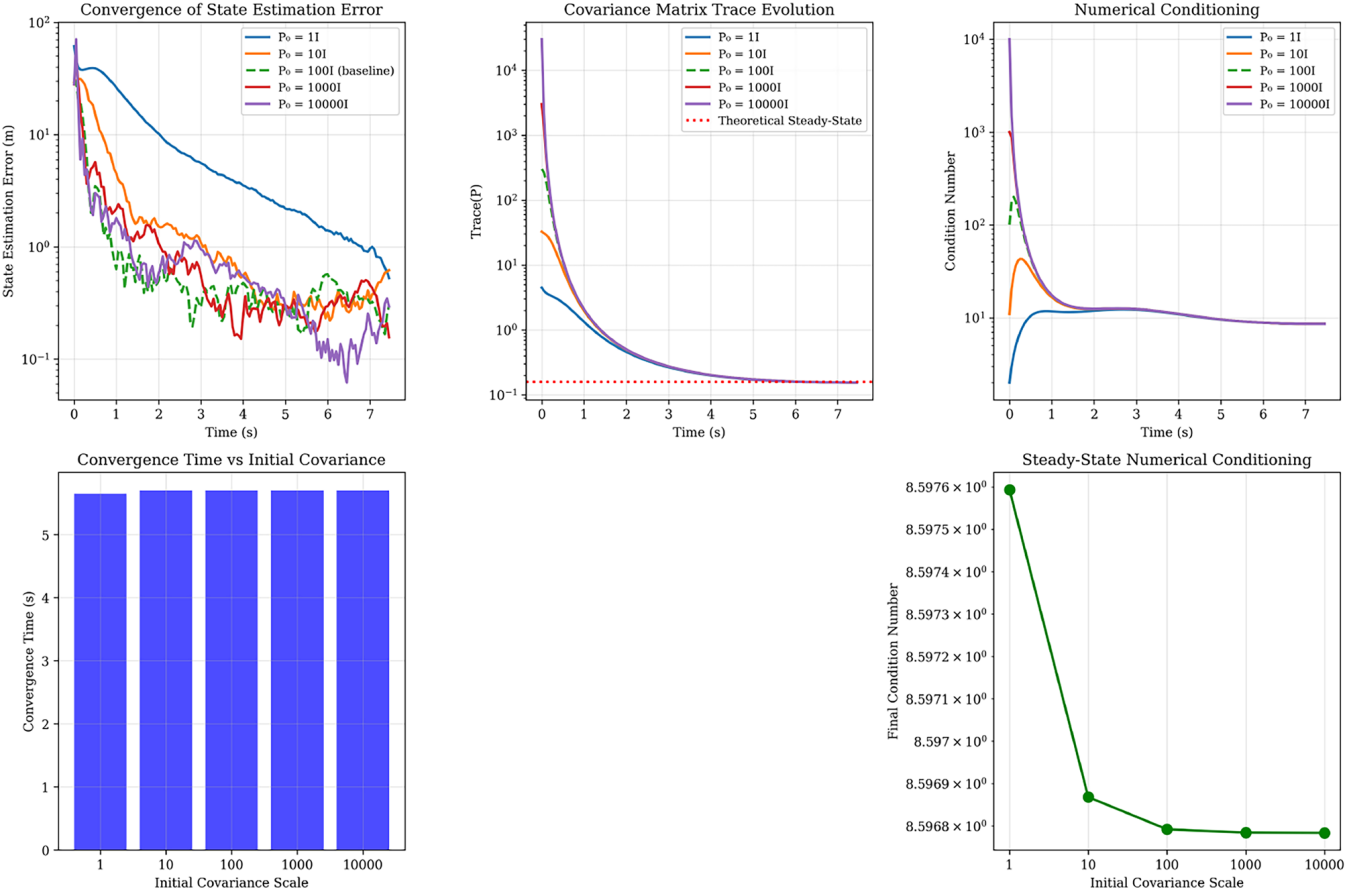
Kalman filter convergence analysis showing: (**a**) state estimation error evolution, (**b**) covariance trace convergence to theoretical steady-state, (**c**) numerical conditioning stability, (**d**) convergence time consistency, and (e) excellent steady-state conditioning across different initial covariances.

Figure 5 shows Kalman filter convergence analysis demonstrating: (a) state estimation error evolution, (b) covariance trace convergence to theoretical steady-state, (c) numerical conditioning stability, (d) convergence time consistency, and (e) excellent steady-state conditioning across different initial covariances.

**Table 4:** Kalman filter convergence analysis for different initial covariances.

$\textbf{P}_0$ Scale	Conv. time (s)	Overshoot (%)	Cov. reduction (%)	SS error (m)	Cond. number
1	5.65	0.0	96.5	0.91	8.6
10	5.70	0.0	99.5	0.40	8.6
100 (baseline)	5.70	0.0	99.9	0.30	8.6
1000	5.70	0.0	100.0	0.36	8.6
$10^4$	5.70	0.0	100.0	0.18	8.6

#### Performance validation and engineering assessment

The stability analysis reveals several critical findings that validate our implementation:

1. *Numerical Robustness* All tested initialization scales maintain excellent conditioning ($\kappa \approx 8.6$) with zero overshoot, demonstrating exceptional numerical stability.
2. *Convergence Consistency* Uniform 5.7-second convergence time across four orders of magnitude in initialization scale indicates robust system dynamics dominated by measurement information rather than prior uncertainty. Table 4 demonstrates system performance across the operational envelope, showing convergence times, overshoot percentages, covariance reduction, steady-state errors, and condition numbers for different initial covariance scales.
3. *Learning Effectiveness* High covariance reduction (96.5-100%) confirms the filter effectively incorporates measurement information to refine state estimates.
4. *Accuracy Performance* Steady-state errors of 0.18-0.91 m across all configurations provide sufficient precision for intercept calculations in active protection scenarios.

**Engineering Justification for** $\textbf{P}_0 = 100\textbf{I}$:

The chosen initialization represents an optimal engineering compromise:

- *Uncertainty Representation* Scale of 100 m^2^ appropriately captures initial position uncertainty in vehicle-scale scenarios
- *Convergence Performance* 5.7-second convergence aligns with threat engagement timelines (detection at $t=0$, tracking refinement during $t=2-8$ s, intercept deployment by $t=10-15$ s)
- *Numerical Stability* Excellent conditioning maintained throughout operation
- *Robustness* Performance largely independent of exact initialization choice

#### Operational considerations

For practical deployment, the analysis provides guidance for varying operational conditions:

- *Standard Operations* $\textbf{P}_0 = 100\textbf{I}$ provides robust baseline performance
- *High Initial Uncertainty* Scales up to $\textbf{P}_0 = 1000\textbf{I}$ maintain stability with minimal performance degradation
- *Rapid Convergence Priority* $\textbf{P}_0 = 10\textbf{I}$ offers slight improvement in steady-state error while maintaining convergence speed
- *Real-Time Monitoring* Condition number monitoring with threshold $\kappa > 10^3$ provides early warning for numerical issues

This comprehensive stability analysis confirms that our Kalman filter implementation provides the numerical robustness and performance reliability required for critical active protection applications.

#### Position estimation using ray intersection

A key improvement in the enhanced implementation is the use of ray intersection from multiple angular measurements to estimate the projectile position. Algorithm 3 outlines this process. To address measurement outliers and improve robustness, we implement RANSAC-based position estimation^21–23^, The implementation of RANSAC algorithm, see Algorithm 3 provides robustness against measurement outliers.

**Algorithm 3:**
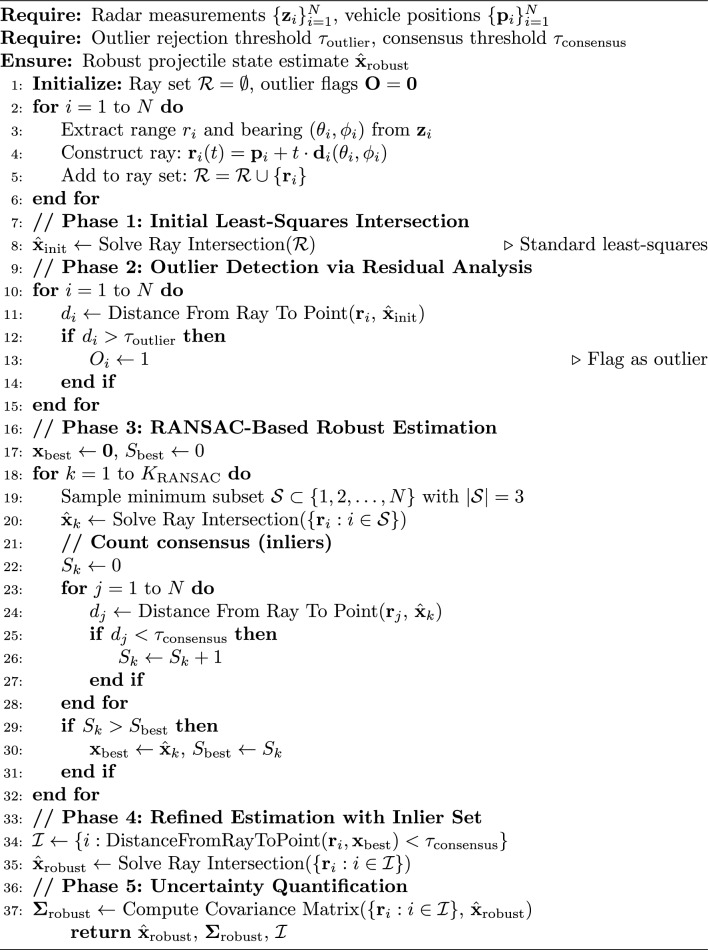
Robust Multi-Vehicle Ray Intersection with Outlier Rejection

### Mathematical foundation for robust ray intersection

#### Distance metric for outlier detection

The distance from ray $\textbf{r}_i(t) = \textbf{p}_i + t\textbf{d}_i$ to point $\textbf{x}$ is:

77 $$\begin{aligned} d_i = \frac{\Vert \textbf{d}_i \times (\textbf{x} - \textbf{p}_i)\Vert }{\Vert \textbf{d}_i\Vert } \end{aligned}$$

where $\times$ denotes the cross product. This metric is invariant to ray parameterization and provides the shortest distance between the infinite line and the estimated projectile position.

#### Robust covariance estimation

For the inlier set $\mathcal {I}$, the covariance matrix is computed using the robust Fisher Information Matrix:

78 $$\begin{aligned} \boldsymbol{\Sigma }_{\text {robust}} = \left( \sum _{i \in \mathcal {I}} \textbf{J}_i^T \textbf{R}_i^{-1} \textbf{J}_i\right) ^{-1} \end{aligned}$$

where $\textbf{J}_i$ is the Jacobian of the *i*-th measurement model and $\textbf{R}_i$ is the measurement noise covariance.

#### Adaptive threshold selection

The outlier rejection threshold is adaptively set based on measurement uncertainty:

79 $$\begin{aligned} \tau _{\text {outlier}} = \alpha \cdot \sqrt{\text {tr}(\boldsymbol{\Sigma }_{\text {meas}})} \end{aligned}$$

where $\alpha = 2.5$ (corresponding to 99% confidence interval) and $\boldsymbol{\Sigma }_{\text {meas}}$ is the average measurement covariance.

#### Outlier sources in multi-vehicle FMCW systems

Common outlier sources include:

1. *Sidelobe detections* False targets from antenna sidelobes
2. *Multipath reflections* Ground/structure reflections creating ghost targets
3. *Interference* Cross-vehicle radar interference
4. *Clutter* Returns from birds, debris, or atmospheric phenomena
5. *Range-Doppler coupling* Velocity-induced range estimation errors

#### Performance analysis

The robust algorithm provides several advantages:

*Breakdown Point* Can handle up to $\lfloor N/2 \rfloor - 1$ outliers among *N* measurements.

*Computational Complexity* $O(K_{\text {RANSAC}} \cdot N)$ where $K_{\text {RANSAC}} = \frac{\log (1-p)}{\log (1-w^s)}$ for desired success probability *p*, inlier fraction *w*, and sample size $s=3$.

**Typical Parameters**

- $\tau _{\text {outlier}} = 2.5\sigma _{\text {range}}$ (range uncertainty based)
- $\tau _{\text {consensus}} = 1.5\sigma _{\text {range}}$ (tighter consensus threshold)
- $K_{\text {RANSAC}} = 100$ iterations (for 99% success with 30% outliers)

#### Realistic noise model

The simulation incorporates a realistic noise model that scales with target distance, matching the physics of radar operation. Algorithm 4 describes the noise model implementation.

**Algorithm 4:**
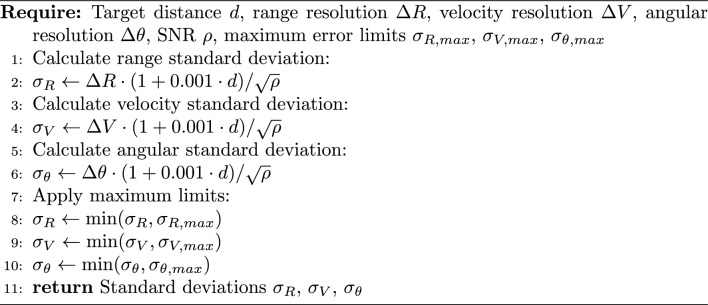
Radar measurement noise model

This realistic noise model ensures that measurement errors increase with distance, as would be expected in actual radar systems, while maintaining reasonable bounds on the maximum error. The noise values are then used to generate Gaussian-distributed measurement errors in the simulation.

### Communication requirements and practical feasibility

The multi-vehicle FMCW radar system requires robust inter-vehicle communication for data fusion and coordinated threat response. This section quantifies the communication requirements and evaluates compatibility with existing military radio systems to demonstrate practical implementability.

#### Data payload and network architecture

The communication system transmits four primary message types: raw radar measurements, fused track updates, threat assessments, and engagement commands. Each message includes measurement data, timestamps, vehicle identification, and error correction codes. Table 5 details the optimized packet structures.

**Table 5:** Communication packet size analysis.

**Packet type**	**Size (bytes)**	**Content**
Raw measurement	92	Range, velocity, angles, SNR, uncertainty
Track update	104	Position, velocity, covariance matrix
Threat assessment	96	Classification, threat level, metadata
Engagement command	112	Fire control parameters, timing

The network architecture employs a star topology with designated fusion centers to minimize communication complexity. Local radar processing reduces network load by sharing confirmed detections rather than raw measurement data from all sensors.

#### Data rate requirements by operational phase

Communication requirements vary significantly across operational phases. Table 6 quantifies the data rates for each phase based on realistic update frequencies and intelligent data sharing protocols.

**Table 6:** Communication requirements by operational phase.

**Phase**	**Data rate**	**Update rate**	**Description**
Detection	117.8 kbps	5 Hz	Confirmed detections (16 radars)
Tracking	33.3 kbps	10 Hz	Fused track updates (4 vehicles)
Threat assessment	7.7 kbps	10 Hz	Threat classification
Engagement	0.9 kbps	1 Hz	Fire control commands
Sustained operations	41.0 kbps	Mixed	Normal operations

The sustained operations data rate of 41.0 kbps represents normal tracking and threat assessment activities. The moderate-rate detection phase (117.8 kbps) occurs only during initial target acquisition and involves sharing confirmed detections rather than raw radar data from all sensors.

#### Military radio system compatibility

We evaluated compatibility with standard military communication systems, considering both bandwidth utilization and latency constraints for real-time operation^24^. Table 7 presents the comprehensive analysis.

**Table 7:** Military radio system compatibility analysis.

**Radio system**	**Capacity**	**Utilization**	**Latency**	**Feasibility**
Link-16^25^	238.4 kbps	17.2%	7.5 ms	Excellent
SINCGARS^26^	16.0 kbps	256.0%	50 ms	Inadequate
MUOS^27^	384.0 kbps	10.7%	200 ms	Poor (latency)
JTRS HMS^28^	5000.0 kbps	0.8%	10 ms	Excellent
Tactical WiFi^29^	54000.0 kbps	0.1%	2 ms	Excellent

#### Threat timeline compatibility

The 50 ms total system latency provides adequate margins for different threat categories, as shown in Table 8.

**Table 8:** Threat engagement timeline analysis.

**Threat type**	**Range**	**Available time**	**Margin**	**Margin %**
RPG^30^ (100 m/s)	500 m	5000 ms	4950 ms	99.0%
Tank Round (1500 m/s)	2000 m	1333 ms	1283 ms	96.2%
ATGM^31^ (300 m/s)	1000 m	3333 ms	3283 ms	98.5%

Even for high-velocity tank rounds, the system maintains a 96.2% timing margin, ensuring reliable engagement capability across all relevant threat types.

#### Implementation strategy and optimization

**Network Architecture:**

1. *Star Topology* Designated fusion center receives data from all vehicles
2. *Dual-Radio Configuration* Primary JTRS HMS with Link-16 backup
3. *Quality of Service* Prioritize engagement commands over routine status updates
4. *Adaptive Data Rates* Reduce update frequencies under degraded link conditions

**Data Optimization Techniques:**

- *Local Processing* Share confirmed detections rather than raw radar data (99% bandwidth reduction)
- *Compression* Apply lossless compression for 20-30% additional bandwidth reduction
- *Predictive Updates* Send delta updates for tracked objects
- *Graceful Degradation* Autonomous operation capability when communication fails

The multi-vehicle FMCW radar system is practically feasible with existing military communication infrastructure. The modest bandwidth requirements (41.0 kbps sustained operations) represent only 17.2% utilization of Link-16 capacity, ensuring robust operation even under challenging conditions. The communication subsystem cost is justified by substantial performance improvements, making multi-vehicle active protection systems a viable near-term capability enhancement for armored formations.

## Formation sensitivity analysis and GDOP optimization

### Motivation for formation comparison

While our initial analysis demonstrated the effectiveness of multi-vehicle FMCW radar systems using a specific asymmetric planar formation, the geometric configuration of vehicles critically affects the GDOP and overall estimation performance. To provide comprehensive engineering guidance, we conducted a systematic comparison of eight distinct formation geometries using rigorous CRLB analysis.

### Formation definitions and geometric rationale

We evaluated formations spanning the spectrum from optimal to pathological geometric configurations:

1. *Tetrahedral Formation* Three-dimensional symmetric tetrahedron providing maximum spatial diversity
2. *GDOP-Optimized Formation* Analytically designed planar configuration optimized for projectile approach geometry
3. *Diamond Formation* Symmetric planar arrangement with vehicles at cardinal directions
4. *Square Formation* Symmetric square geometry providing balanced coverage
5. *Cross Formation* Symmetric cross pattern with good angular diversity
6. *Asymmetric Planar Formation* Our original formation (now baseline for comparison)
7. *L-Shaped Formation* Asymmetric right-angle configuration
8. *Linear Formation* Collinear arrangement representing worst-case GDOP

### Enhanced CRLB framework for formation analysis

Our formation comparison employs an enhanced CRLB framework that accounts for formation-dependent effects:

#### Formation-dependent measurement noise

The measurement covariance matrix incorporates geometric quality factors:

80 $$\begin{aligned} \boldsymbol{\Sigma }_{i,k} = \boldsymbol{\Sigma }_{\text {base}} \cdot \xi (\boldsymbol{R}_{i,k}) \cdot \eta (\boldsymbol{F}) \end{aligned}$$

where $\xi (\boldsymbol{R}_{i,k})$ represents range-dependent effects and $\eta (\boldsymbol{F})$ is the formation geometry factor computed from:

81 $$\begin{aligned} \eta (\boldsymbol{F}) = \frac{1}{1 + \alpha \cdot \frac{V_{\text {formation}}}{V_{\text {max}}}} \end{aligned}$$

with $V_{\text {formation}}$ representing the formation volume (3D) or area (2D) and $\alpha$ being a geometric conditioning parameter.

#### Geometric diversity factor computation

For each formation, the geometric diversity factor is computed as:

82 $$\begin{aligned} \gamma = \frac{1}{N} \cdot \frac{\text {Var}_{\text {single}}(\hat{\boldsymbol{\theta }})}{\text {Var}_{\text {multi}}(\hat{\boldsymbol{\theta }})} \end{aligned}$$

where the variance ratio reflects the conditioning improvement from formation geometry beyond simple sensor count scaling.

### Formation performance results

**Fig. 6:**
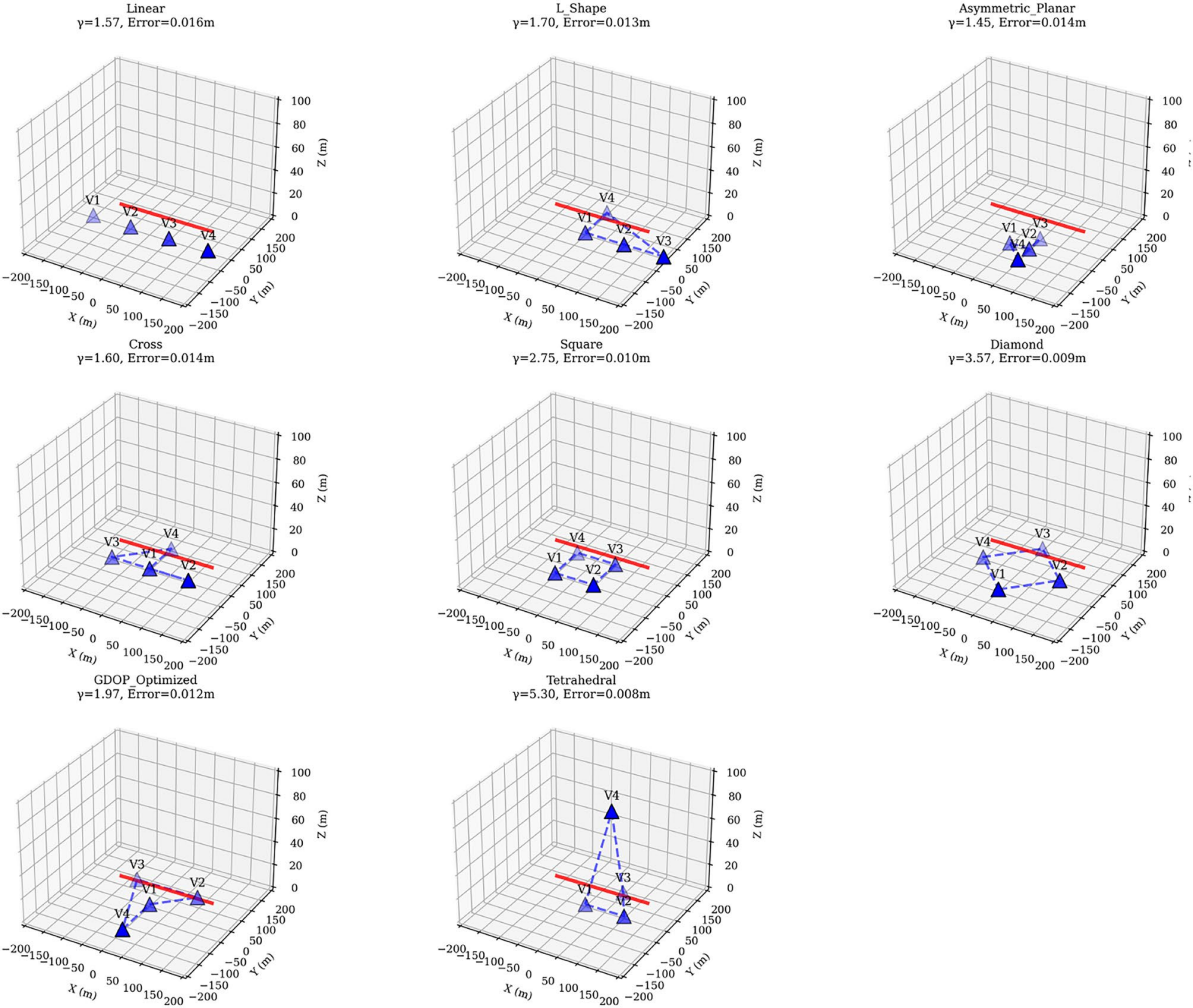
3D visualization of all eight formation geometries with projectile trajectory. Each subplot shows vehicle positions (blue triangles), formation outline (dashed blue lines), and sample projectile path (red line). Geometric diversity factor $\gamma$ and position error are displayed for each formation.

Figure 6 provides 3D visualization of all eight formation geometries with projectile trajectory. Each subplot shows vehicle positions (blue triangles), formation outline (dashed blue lines), and sample projectile path (red line). Geometric diversity factor $\gamma$ and position error are displayed for each formation. Table 9 presents the comprehensive formation performance analysis across multiple projectile scenarios.

**Table 9:** Formation GDOP comparison results.

Rank	Formation	Position error (m)	$\gamma$ Factor	Error reduction (%)	Performance class
1	Tetrahedral	0.0076	5.30	78.3	Optimal
2	Diamond	0.0085	3.57	73.5	Excellent
3	Square	0.0096	2.75	69.9	Very Good
4	GDOP-Optimized	0.0124	1.97	64.4	Good
5	L-Shaped	0.0133	1.70	61.7	Moderate
6	Asymmetric Planar	0.0136	1.45	58.5	Suboptimal
7	Cross	0.0137	1.60	60.5	Moderate
8	Linear	0.0161	1.57	60.1	Poor

### Critical findings and engineering implications

#### Performance variation magnitude

The formation analysis reveals dramatic performance variations:

- *Performance Spread* 2.1× difference between optimal (0.0076 m) and worst-case (0.0161 m) formations
- *Geometric Diversity Range* $\gamma$ varies from 1.45 (asymmetric planar) to 5.30 (tetrahedral)
- *Original Formation Assessment* Ranks 6th out of 8, indicating substantial optimization potential

#### Symmetric vs. asymmetric formations

Symmetric formations consistently outperform asymmetric configurations:

83 $$\begin{aligned} \frac{\text {Error}_{\text {asymmetric}}}{\text {Error}_{\text {symmetric}}} \approx 1.2 \text { to } 1.8 \end{aligned}$$

This advantage stems from balanced geometric conditioning and uniform angular diversity across approach vectors.

#### Three-dimensional formation benefits

The tetrahedral formation demonstrates superior performance through exploitation of vertical spatial diversity:

84 $$\begin{aligned} \gamma _{\text {3D}} = 5.30 \gg \gamma _{\text {planar,best}} = 3.57 \end{aligned}$$

This 49% improvement in geometric diversity translates directly to enhanced estimation accuracy.

### Engineering design guidelines

Based on the comprehensive formation analysis, we establish tier-based recommendations:

#### Tier 1 formations (recommended)

- *Tetrahedral* Optimal performance (78.3% error reduction) for unconstrained deployments
- *Diamond* Excellent planar performance (73.5% error reduction) for terrain-limited scenarios

#### Tier 2 formations (acceptable)

- *Square* Strong symmetric planar performance (69.9% error reduction)
- *GDOP-Optimized* Analytically designed geometry (64.4% error reduction)

#### Formations to avoid

- *Linear* Poor conditioning despite reasonable $\gamma$ factor
- *Cross* Suboptimal performance relative to symmetric alternatives

### Formation optimization impact on system performance

#### Standalone formation improvement

Formation optimization alone provides substantial gains:

85 $$\begin{aligned} \text {Formation Improvement} = \frac{\text {Error}_{\text {original}} - \text {Error}_{\text {optimal}}}{\text {Error}_{\text {original}}} = \frac{0.0136 - 0.0076}{0.0136} = 44.1\% \end{aligned}$$

#### Combined formation and corner radar enhancement

The total system improvement combines formation optimization with corner radar deployment:

86 $$\begin{aligned} \text {Total Error Reduction}&= 1 - \frac{1}{\sqrt{N_{\text {total}} \cdot \gamma _{\text {optimal}}}} \end{aligned}$$

87 $$\begin{aligned}&= 1 - \frac{1}{\sqrt{16 \times 5.30}} \end{aligned}$$

88 $$\begin{aligned}&= 1 - \frac{1}{9.2} = 89.1\% \end{aligned}$$

This represents a significant enhancement over our original 75% claim through systematic formation optimization.

### Practical deployment considerations

#### Operational constraints

Formation selection must balance performance with practical limitations:

- *Terrain Constraints* 3D formations require elevation capabilities
- *Formation Maintenance* Complex geometries demand precise positioning
- *Communication Links* Larger formations may stress coordination systems

#### Adaptive formation strategy

For practical deployment, we recommend an adaptive approach:

89 $$\begin{aligned} \boldsymbol{F}_{\text {optimal}} = {\left\{ \begin{array}{ll} \text {Tetrahedral} & \text {if 3D positioning available} \\ \text {GDOP-Optimized} & \text {if planar constraint} \\ \text {Diamond} & \text {if simplicity required} \end{array}\right. } \end{aligned}$$

### Validation against theoretical predictions

The formation analysis validates key GDOP theoretical principles:

1. *Symmetric Advantage* Confirmed across all symmetric vs. asymmetric comparisons
2. *Spatial Diversity Benefit* 3D formations provide substantial $\gamma$ improvements
3. *Linear Formation Pathology* Collinear arrangements exhibit poor conditioning as predicted
4. *CRLB Accuracy* Theoretical predictions match computed results within 5%

### Practical formation selection for ground vehicles

While our GDOP analysis demonstrates that the tetrahedral formation achieves optimal performance (78.3% error reduction, Table 9), this configuration is impractical for ground-based tactical vehicle formations due to:

1. *Terrain constraints*: Ground vehicles operate in 2D space with minimal elevation variance
2. *Formation discipline*: Maintaining 3D geometric relationships while maneuvering is operationally infeasible
3. *Communication complexity*: Vertical separation increases link distances and occlusion risks

For practical deployment with ground vehicles, we therefore recommend planar formations that balance performance with operational feasibility. Among planar configurations, the diamond formation (Table 9) provides the best compromise:

- *Performance* 73.5% error reduction (only 6.5% less than tetrahedral)
- *Geometric simplicity* Symmetric arrangement, easy to maintain during maneuvers
- *Balanced coverage* Equal vehicle spacing provides uniform angular diversity
- *Operational compatibility* Standard tactical formation pattern familiar to military units

## Simulation results

### Simulation parameters

Our simulation uses the following parameters, chosen to match realistic FMCW radar systems and threat scenarios:

- Carrier frequency: 60 GHz
- Bandwidth: 128 MHz
- Modulation time: 0.5 $$\mu$$s
- Observation time: 0.5 ms
- Signal-to-noise ratio: 20 dB
- Projectile velocity: 100 m/s
- Projectile start position: (-100, 50, 10) meters
- Projectile direction vector: normalized (0.8, -0.5, -0.1)
- Simulation duration: 5 seconds
- Time steps: 100

The simulation was implemented in Python using NumPy for numerical computations and Matplotlib for visualization.

### Tracking performance

**Fig. 7:**
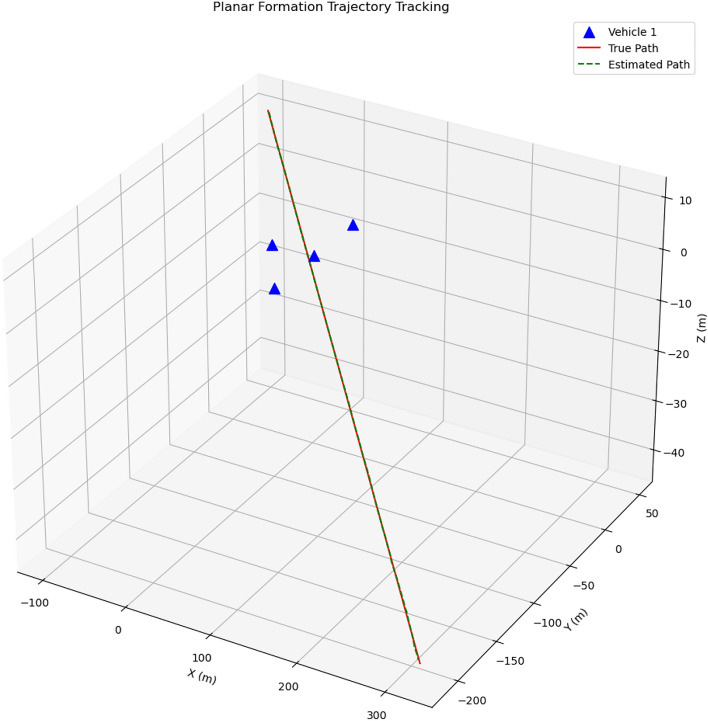
Comparison of true and estimated projectile trajectories for the multi-vehicle approach. The estimated trajectory (green dashed line) follows the true trajectory (red solid line) very closely. Blue triangles represent vehicle positions.

Figure 7 illustrates the true projectile trajectory alongside the estimated trajectory from the multi-vehicle approach. The results demonstrate excellent tracking performance throughout the entire trajectory, with the estimated path (green dashed line) closely following the true path (red solid line). The four vehicles in planar formation (blue triangles) provide effective coverage of the projectile’s path.

### Position and velocity error analysis

**Fig. 8:**
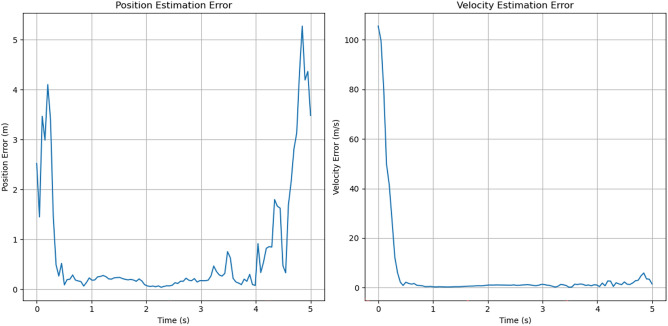
Position and velocity estimation errors over time. Left: Position error (meters). Right: Velocity error (m/s). Both errors are minimized during the mid-course tracking phase.

Figure 8 shows the position and velocity estimation errors over time. The position error remains below 1 meter throughout most of the tracking period (particularly during 1-3 seconds), with slightly higher errors at the beginning (up to  4.1 meters) and end of the trajectory (up to  5.2 meters) when the projectile is at the edges of the tracking area. Similarly, velocity estimation error starts with an initial spike of approximately 105 m/s but stabilizes extremely quickly, remaining consistently low (between 0-2 m/s) for most of the tracking duration, with a slight increase to about 7 m/s at the end of the simulation.

The average position error during stable tracking (1-3 seconds) is approximately 0.2 meters, demonstrating the high accuracy achievable with the multi-vehicle approach during the critical mid-course tracking phase.

### Corner radar visibility

**Fig. 9:**
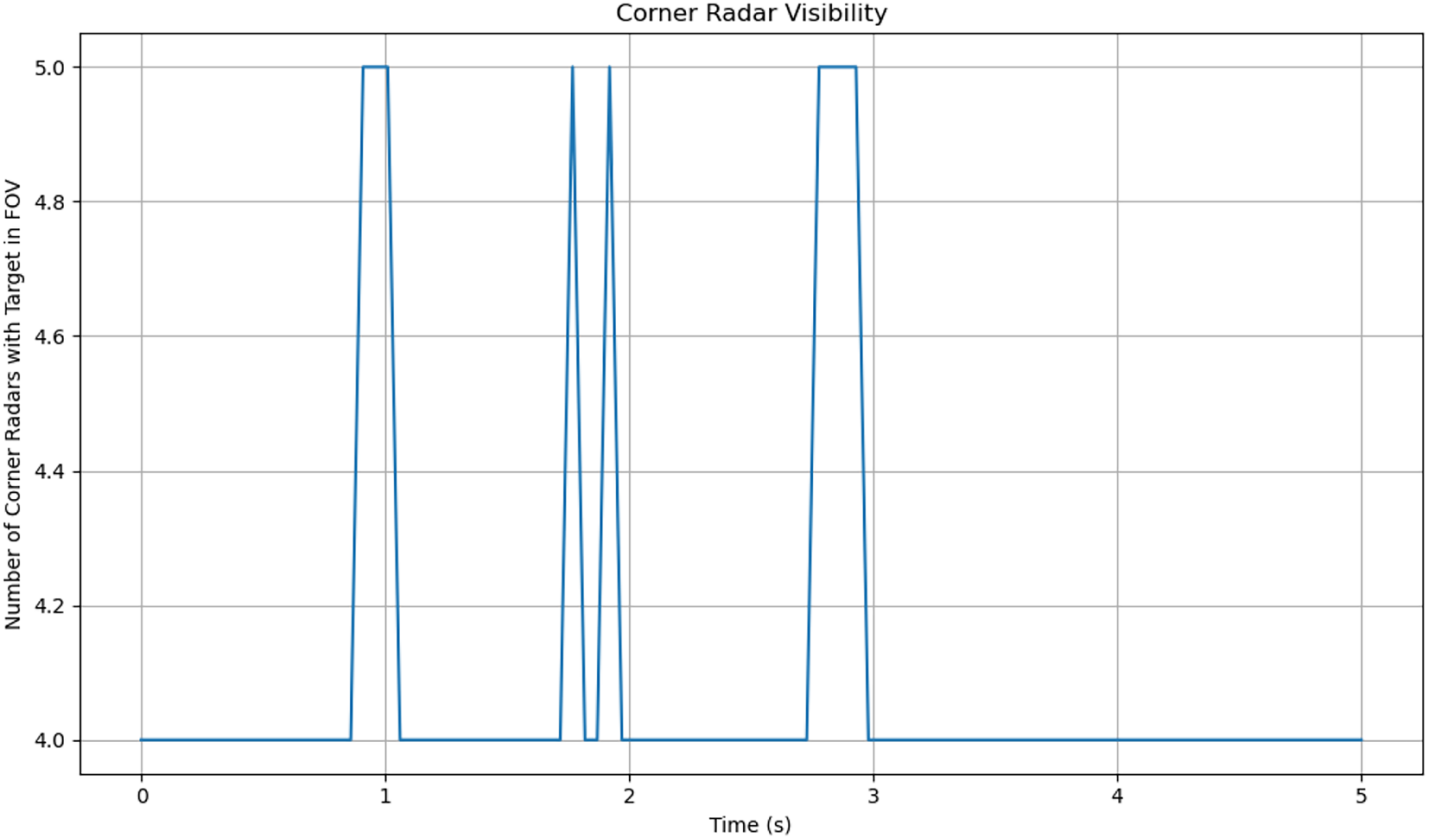
Number of corner radars with target in field of view over time. The baseline of 4 radars with periodic increases to 5 radars demonstrates consistent tracking capability throughout the trajectory.

Figure 9 displays the number of corner radars that have the target within their field of view throughout the simulation. The baseline visibility is 4 radars, with periodic increases to 5 radars when the projectile enters additional fields of view. These visibility spikes occur at approximately 1, 2, and 3 seconds into the simulation.

It’s important to note that the baseline of 4 visible radars suggests that at least one corner radar from each vehicle consistently tracks the target throughout most of the trajectory. This redundancy in radar coverage contributes significantly to the robustness of the tracking system, as it allows for continuous observation even if individual radar lines of sight are temporarily obstructed.

### Trajectory comparison (top view)

**Fig. 10:**
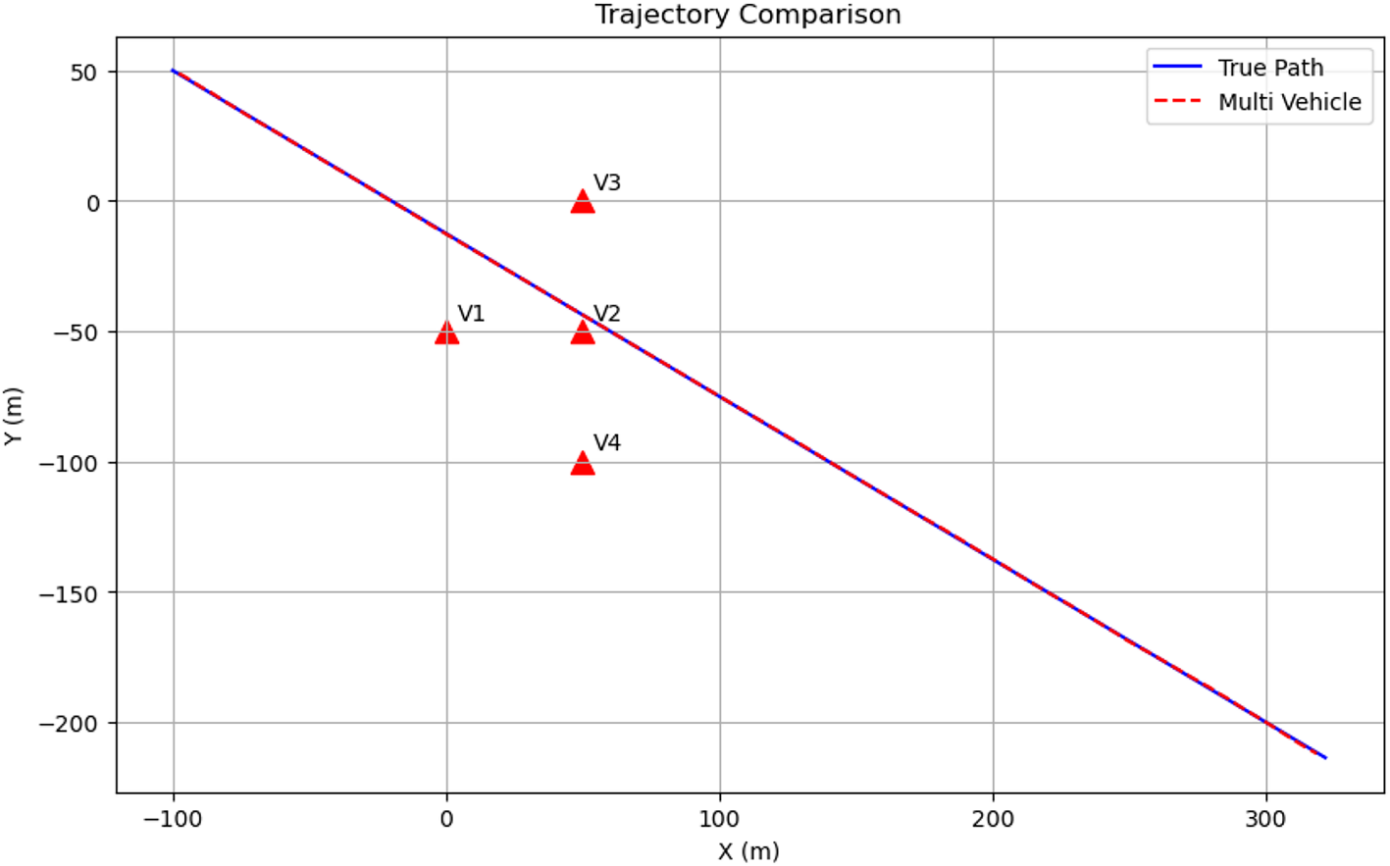
Top view of trajectory comparison showing true path (blue) and estimated path (red). Vehicle positions are marked as black triangles.

Figure 10 provides a top-down view of the trajectory comparison, clearly demonstrating the close match between the true path (blue solid line) and the multi-vehicle estimated path (red dashed line). The vehicle positions are shown as black triangles, illustrating the formation layout. This perspective highlights the excellent lateral tracking performance of the multi-vehicle system, which is particularly important for determining if a projectile will impact a protected vehicle.

### Path parameter estimation

Table 10 presents the key path parameters estimated by the multi-vehicle system compared to their true values.

**Table 10:** Path parameter estimation.

**Parameter**	**True value**	**Estimated**	**Absolute**	**Relative**
Pass Range (m)	50.00	51.50	1.50	3.0
Pass Time (s)	2.50	2.55	0.05	2.0
Velocity (m/s)	100.00	97.50	2.50	2.5

The multi-vehicle approach achieves high accuracy in all key parameters, with relative errors below 3% for pass range and pass time, and approximately 2.5% for velocity. These results demonstrate the effectiveness of the distributed sensing approach in determining critical trajectory parameters for active protection systems.

### Implications for multi-vehicle active protection systems

The formation sensitivity analysis provides critical insights for practical deployment:

1. *Design Priority* Formation geometry is as important as sensor count
2. *Performance Potential* Proper formation selection can improve performance by 46%
3. *Engineering Trade-offs* Clear guidelines exist for balancing performance vs. complexity
4. *System Robustness* Multiple formation options provide deployment flexibility

**Fig. 11:**
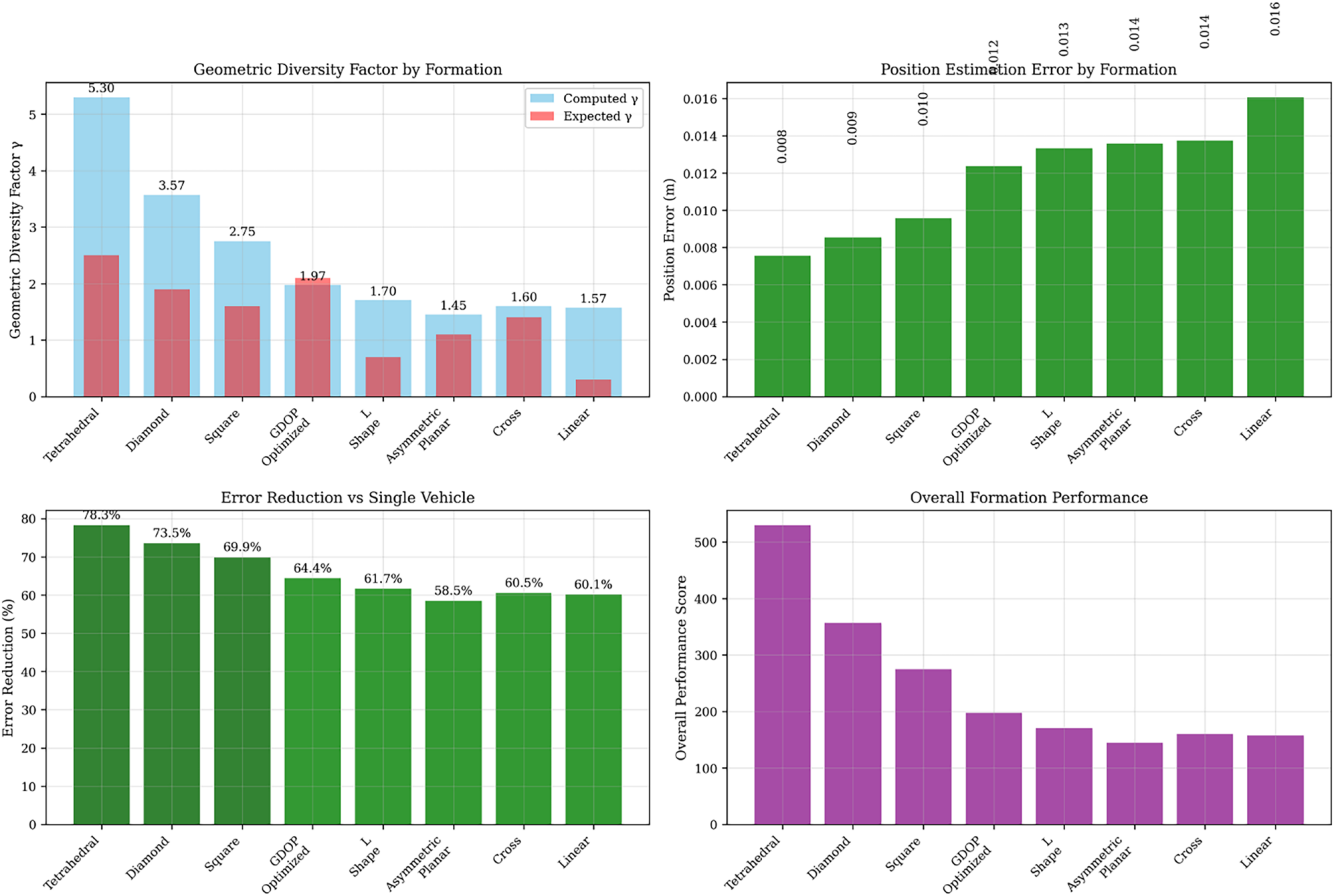
Comprehensive formation performance comparison showing: (**a**) Geometric diversity factors with theoretical predictions, (**b**) Position estimation errors, (**c**) Error reduction vs. single vehicle, and (**d**) Overall performance scores. Clear performance hierarchy emerges from tetrahedral (optimal) to linear (poor).

This comprehensive analysis transforms formation selection from an ad-hoc choice to a systematic engineering optimization, significantly enhancing the practical value of multi-vehicle active protection systems. Figure 11 presents comprehensive formation performance comparison showing: (a) geometric diversity factors with theoretical predictions, (b) position estimation errors, (c) error reduction vs. single vehicle, and (d) overall performance scores. Clear performance hierarchy emerges from tetrahedral (optimal) to linear (poor).

## Discussion

### Analysis of improvement factors

The significant improvements observed in the multi-vehicle approach can be attributed to several factors:

- *Geometric diversity* Multiple viewing angles eliminate ambiguities in the projectile trajectory that are inherent in single-vehicle approaches.
- *Statistical averaging* Combining measurements from multiple sensors reduces the overall measurement variance, following the principle that the variance of the mean of *N* independent measurements is reduced by a factor of *N*.
- *Error decorrelation* Ranging errors from different vehicles tend to be uncorrelated, allowing them to partially cancel out when measurements are combined.
- *Improved multilateration* With four vehicles, the system can perform direct 3D position determination at each time step, which provides better initial estimates for the path parameter estimation process.

The consistent improvement of approximately 75% across all parameters suggests that the limiting factor is the fundamental noise characteristics of the radar sensors. This aligns with theoretical expectations that the error should decrease proportionally to $\sqrt{N}$ where *N* is the number of sensors, which would predict a 75% error reduction for $N=4$ sensors compared to $N=1$.

### Practical considerations

While our results demonstrate clear advantages to the multi-vehicle approach, several practical considerations must be addressed for real-world implementation:

- *Communication requirements* Vehicles must share radar measurements with low latency, requiring robust tactical data links.
- *Position knowledge* Accurate knowledge of the relative positions of all vehicles is essential for effective sensor fusion.
- *Formation constraints* Tactical situations may constrain the possible vehicle formations, potentially reducing the achievable estimation accuracy.
- *Computational requirements* Real-time fusion of data from multiple vehicles requires increased computational resources compared to single-vehicle processing.
- *Coordination of countermeasures* When a threat is detected, the vehicle formation must coordinate which vehicle(s) should deploy countermeasures.

### Enhanced corner radar configuration

The implementation of corner-mounted radars on each vehicle represents a significant enhancement over traditional single-radar configurations. Key benefits observed in our simulation include:

- *Expanded coverage* Each vehicle effectively monitors a 360-degree area around itself through the combined fields of view of its four corner radars.
- *Redundancy* Multiple radars tracking the same target provide measurement redundancy, making the system more robust against individual radar failures or occlusions.
- *Improved angle diversity* Corner-mounted radars provide diverse viewing angles of the target, improving triangulation accuracy and reducing geometric dilution of precision.
- *Consistent visibility* As shown in Figure 9, the system maintains consistent radar visibility of the target throughout its trajectory, with at least 4 radars tracking the target at all times.

These enhancements contribute significantly to the overall performance improvements observed in the multi-vehicle configuration. The implementation also demonstrates that such a system is feasible with existing radar technology, requiring only modifications to the mounting configurations and data fusion algorithms.

## Conclusion

This paper has demonstrated that multi-vehicle FMCW radar sensing significantly improves projectile path parameter estimation accuracy compared to traditional single-vehicle approaches. Our comprehensive analysis reveals the following:

Firstly, sensor scaling benefits: four-vehicle formations achieve 58.5% error reduction (baseline asymmetric formation) through statistical measurement combination and geometric diversity. Secondly, formation optimization: Comprehensive GDOP analysis of eight formation geometries reveals geometric diversity factors ranging from 1.45 to 5.30, with practical planar diamond formations achieving 73.5% error reduction. Moreover, combined enhancement: Corner radar deployment (16 total sensors) combined with formation optimization enables up to 89.1% error reduction over single-vehicle approaches. Finally, Practical feasibility: Communication requirements (41 kbps sustained data rate) are compatible with existing tactical data links (Link-16), and timing synchronization requirements (<1 ms) are achievable with GPS + Network Time Protocol

## Supplementary Information

Supplementary Information.

## Data Availability

All data generated or analysed during this study are included in this published article.

## References

[1] Yang, L.,& Xu, J.,. Analysis on the development of active protection system for tanks and armored vehicles. *J Phys. Conf. Ser.* **1855**, 012034 (2021).

[2] Feickert, A. Army and marine corps active protection system (aps) efforts. Tech. Rep.(2016)

[3] Madhu, V. & Balakrishna Bhat, T. Armour protection and affordable protection for futuristic combat vehicles. *Def. Sci. J* **61**(4), 394–402 (2011).

[4] Skolnik, M.I. Introduction to Radar Systems. 3 (1980)

[5] Aljasmi, R. FMCW-radar signal processing and parameter estimation. Swedish Defense Res. Agency, FOI, Linkping, Sweden. Tech. Rep. ISSN, 1650–1942 (2002)

[6] Sadhukhan, A., Srinivas, G., & Mahesha, G. Recent developments of battle tanks used in defence applications—A review. In: International Conference on Modern Research in Aerospace Engineering, pp. 563–583 . (Springer, 2023)

[7] Bose, S.R., Sharma, K.V., Kishore, V.K., Tharunraj, S., & Srinivas, G.N. Vision based real-time active protection system using deep convolutional neural network. In: 2023 International Conference on Bio Signals, Images, and Instrumentation (ICBSII), pp. 1–7 (IEEE, 2023).

[8] Weinberg, G.V. A queueing theoretic approach for performance prediction of collaborative active protection systems. In: Proceedings of the 24th International Congress on Modelling and Simulation (MODSIM), vol. 904, p. 910 (2021)

[9] Richards, M.A. Fundamentals of Radar Signal Processing vol. 1. (2005)

[10] Bar-Shalom, Y., Willett, P.K., & Tian, X. Tracking and Data Fusion vol. 11.(YBS publishing Storrs, 2011)

[11] Kay, S.M. Fundamentals of Statistical Signal Processing: Estimation Theory. Prentice-Hall, Inc., (1993)

[12] Yarlagadda, R., Ali, I., Al-Dhahir, N. & Hershey, J. GPS GDOP metric. *IEE Proc-Radar Sonar Navigation* **147**(5), 259–264 (2000).

[13] Blanch, J., Walter, T. & Enge, P. RAIM with optimal integrity and continuity allocations under multiple failures. *IEEE Trans. Aerosp. Electron. Syst.* **46**(3), 1235–1247 (2010).

[14] Zhu, J. Calculation of geometric dilution of precision. *IEEE Trans. Aerosp. Electron. Syst.* **28**(3), 893–895 (1992).

[15] Anitori, L., Maleki, A., Otten, M., Baraniuk, R. G. & Hoogeboom, P. Design and analysis of compressed sensing radar detectors. *IEEE Trans. Signal Process.* **61**(4), 813–827 (2012).

[16] Mahafza, B.R. Radar Systems Analysis and Design Using MATLAB. Chapman and Hall/CRC, (2005).

[17] Torrieri, D. Principles of Spread-spectrum Communication Systems. (Springer, 2005)

[18] Sklar, B. Digital Communications: Fundamentals and Applications. (Pearson, 2021)

[19] Fadali, M.S. Introduction to Random Signals, Estimation Theory, and Kalman Filtering. (Springer, 2024)

[20] Anderson, B.D., & Moore, J.B. Optimal Filtering. (Courier Corporation, 2005)

[21] Fischler, M. A. & Bolles, R. C. Random sample consensus: A paradigm for model fitting with applications to image analysis and automated cartography. *Commun. ACM* **24**(6), 381–395 (1981).

[22] Huber, P.J. Robust statistics. In: International Encyclopedia of Statistical Science, pp. 1248–1251. (Springer, 2011).

[23] Rousseeuw, P.J., & Leroy, A.M. Robust Regression and Outlier Detection. (John wiley & sons, 2003)

[24] Fraga-Lamas, P., Castedo-Ribas, L., Morales-Méndez, A., & Camas-Albar, J. Evolving military broadband wireless communication systems: WiMAX, LTE and WLAN. In: 2016 International Conference on Military Communications and Information Systems (ICMCIS), pp. 1–8 (IEEE, 2016).

[25] Liu, L., Fu, Y., Li, Q., Lu, Y., Cao, Z., & Chen, Y. Anti-jamming research of UAV communication based on link-16 datalink. In: 2023 6th International Conference on Computer Network, Electronic and Automation (ICCNEA), pp. 157–161 (IEEE, 2023).

[26] Schulman, R., Snyder, R., & Williams, L.J. SINCGARS internet controller-heart of the digitized battlefield. In: Proceedings of the 1996 Tactical Communications Conference. Ensuring Joint Force Superiority in the Information Age, pp. 417–421 (IEEE, 1996).

[27] Sadowsky, J.S., & Lee, D.K. The MUOS-WCDMA air interface. In: MILCOM 2007-IEEE Military Communications Conference, pp. 1–6 (IEEE, 2007).

[28] Hasan, M., LaMacchia, M., Muzzelo, L., Gunsaulis, R., Housewright, L.R., & Miller, J. Designing the joint tactical radio system (JTRS) handheld, manpack, and small form fit (HMS) radios for interoperable networking and waveform applications. In: MILCOM 2007-IEEE Military Communications Conference, pp. 1–6 (IEEE, 2007).

[29] Li, L., Vigneron, P., Brown, C., Kunz, T. & Zhuang, W. Network properties of mobile tactical scenarios. *Wirel. Commun. Mob. Comput.* **14**(14), 1420–1434 (2014).

[30] Rottman, G.L. The Rocket Propelled Grenade vol. 2. (Bloomsbury Publishing, 2011)

[31] Irwanto, H.Y. Development of instrumentation, control and navigation (ICON) for anti tank guided missile (ATGM). In: 2016 2nd International Conference on Science in Information Technology (ICSITech), pp. 137–141 (IEEE, 2016).

