# Supplementary material for: Enhanced projectile path estimation using multi-vehicle FMCW radar sensors
Source: Sci Rep. 2026 Feb 2;16:4533. doi: 10.1038/s41598-025-20772-6 (PMC12867980; doi:10.1038/s41598-025-20772-6)
Supplement: Supplementary file 1 — Supplementary Information. [file 41598_2025_20772_MOESM1_ESM.pdf]

Appendix A Spatial Correlation and Multipath Effects in Multi-Vehicle FMCW Radar

The CRLB analysis in Section **??** assumes independent measurement noise between spatially separated radars. However, for closely spaced corner radars and in realistic propagation environments, this assumption may be violated due to spatial correlation and multipath effects. This appendix provides a comprehensive theoretical framework incorporating these effects using both ray-tracing and stochastic channel models.

A.1 Correlated Noise Model for Corner Radars

For the enhanced corner radar configuration with $M = 16$ radars, the measurement vector becomes:

$$\boldsymbol{z}_k = \boldsymbol{h}(\boldsymbol{\theta}, t_k) + \boldsymbol{w}_k \tag{A1}$$

where $\boldsymbol{w}_k \sim \mathcal{N}(\mathbf{0}, \boldsymbol{\Sigma}_k)$ with correlated covariance matrix $\boldsymbol{\Sigma}_k$.

A.1.1 Spatial Correlation Function

The correlation between radars i and j separated by distance d_{ij} is modeled using the exponential correlation function:

$$\rho_{ij}(d_{ij}) = \exp\left(-\frac{d_{ij}}{d_c}\right)\cos\left(\frac{2\pi d_{ij}}{\lambda}\right) \tag{A2}$$

where d_c is the correlation distance and λ is the radar wavelength.

For 60 GHz FMCW radar:

$$\lambda = \frac{c}{f_c} = \frac{3 \times 10^8}{60 \times 10^9} = 5 \text{ mm} \tag{A3}$$

$$d_c \approx 10\lambda = 50 \text{ mm} \tag{A4}$$

A.1.2 Corner Radar Correlation Matrix

For corner radars mounted on vehicle i with separation $d_{\text{corner}} = 4$ m (diagonal distance), the correlation coefficient is:

$$\rho_{\text{corner}} = \exp\left(-\frac{4}{0.05}\right)\cos\left(\frac{2\pi \times 4}{0.005}\right) \approx 0 \tag{A5}$$

However, for same-side radars with $d = 2\sqrt{2}$ m separation:

$$\rho_{\text{side}} = \exp\left(-\frac{2\sqrt{2}}{0.05}\right)\cos\left(\frac{2\pi \times 2\sqrt{2}}{0.005}\right) \approx 0 \tag{A6}$$

This analysis confirms that corner radars are sufficiently separated to maintain measurement independence.

A.2 Ray-Tracing Model for Multipath Effects

A.2.1 Multipath Channel Model

The received signal at radar i includes both direct path and multipath components:

$$s_i(t) = s_{\text{direct},i}(t) + \sum_{p=1}^{P} s_{\text{multipath},i,p}(t) \tag{A7}$$

For ground-based vehicles, the dominant multipath component is ground reflection:

$$s_{\text{ground},i}(t) = \Gamma \cdot s_{\text{direct},i}(t - \tau_{\text{ground},i}) \cdot e^{j\phi_{\text{ground},i}} \tag{A8}$$

where Γ is the ground reflection coefficient and $\tau_{\text{ground},i}$ is the additional delay.

A.2.2 Ground Reflection Geometry

For a projectile at height h_p and vehicle at height h_v, the additional path length due to ground reflection is:

$$\Delta R_{\text{ground}} = \sqrt{R_{\text{direct}}^2 + 4h_p h_v} - R_{\text{direct}} \tag{A9}$$

For typical scenarios ($h_p = 10$ m, $h_v = 2$ m, $R_{\text{direct}} = 500$ m):

$$\Delta R_{\text{ground}} = \sqrt{500^2 + 4 \times 10 \times 2} - 500 \approx 0.16 \text{ m} \tag{A10}$$

A.2.3 Multipath-Induced Range Error

The range measurement bias due to multipath is:

$$\epsilon_{\text{multipath}} = \frac{\Gamma \Delta R_{\text{ground}}}{1 + \Gamma \cos(\phi_{\text{ground}})} \tag{A11}$$

For typical ground reflection ($\Gamma = 0.3$, $\phi_{\text{ground}} = \pi$):

$$\epsilon_{\text{multipath}} = \frac{0.3 \times 0.16}{1 + 0.3 \times (-1)} = 0.069 \text{ m} \tag{A12}$$

This bias is small compared to the range resolution ($\sigma_R \approx 1.17$ m) and can be mitigated through diversity.

A.3 Stochastic Channel Model

A.3.1 Rician Fading Model

The received signal amplitude follows a Rician distribution in the presence of multipath:

$$|h_i|^2 \sim \text{Rician}(K_i, \Omega_i) \tag{A13}$$

where K_i is the Rician K-factor and Ω_i is the total received power.

For line-of-sight dominated scenarios typical in active protection systems:

$$K_i = \frac{P_{\text{LOS}}}{P_{\text{multipath}}} \approx 10 \text{ dB} \tag{A14}$$

A.3.2 Correlated Rician Channels

The correlation between channels i and j in a Rician environment is:

$$\rho_{ij}^{\text{Rician}} = \frac{\sqrt{K_i K_j}}{(1+K_i)(1+K_j)} + \frac{\rho_{ij}^{\text{Rayleigh}}}{\sqrt{(1+K_i)(1+K_j)}} \tag{A15}$$

For high K-factor scenarios ($K > 10$ dB), the correlation is dominated by the line-of-sight component, reducing correlation between spatially separated radars.

A.4 Modified Fisher Information Matrix

A.4.1 Correlated Measurement Covariance

The measurement covariance matrix incorporating spatial correlation becomes:

$$[\mathbf{\Sigma}_k]_{ij} = \begin{cases} \sigma_i^2 & \text{if } i = j \\ \rho_{ij}\sigma_i\sigma_j & \text{if } i \neq j \end{cases} \tag{A16}$$

For corner radars with negligible correlation ($\rho_{ij} \approx 0$ for $d_{ij} > 2$ m), this reduces to the diagonal case used in the main analysis.

A.4.2 Modified CRLB with Correlation

The Fisher Information Matrix with correlated measurements is:

$$\boldsymbol{F}_k = \boldsymbol{H}_k^T \mathbf{\Sigma}_k^{-1} \boldsymbol{H}_k \tag{A17}$$

For block-diagonal correlation structure (correlation within vehicles, independence between vehicles):

$$\mathbf{\Sigma}_k^{-1} = \text{blockdiag}(\mathbf{\Sigma}_{1,k}^{-1}, \mathbf{\Sigma}_{2,k}^{-1}, \mathbf{\Sigma}_{3,k}^{-1}, \mathbf{\Sigma}_{4,k}^{-1}) \tag{A18}$$

A.5 Numerical Analysis of Correlation Effects

A.5.1 Effective Number of Independent Measurements

When measurements are correlated, the effective number of independent measurements is:

$$N_{\text{eff}} = \frac{(\text{tr}(\mathbf{\Sigma}))^2}{\text{tr}(\mathbf{\Sigma}^2)} \tag{A19}$$

For the corner radar configuration with 4 radars per vehicle:

- Uncorrelated case: $N_{\text{eff}} = 16$
- Moderate intra-vehicle correlation ($\rho = 0.3$): $N_{\text{eff}} = 13.2$
- Strong intra-vehicle correlation ($\rho = 0.7$): $N_{\text{eff}} = 8.8$

A.5.2 Performance Degradation Analysis

The performance degradation due to correlation can be quantified as:

$$\eta_{\text{correlation}} = \frac{\sqrt{N_{\text{eff}}}}{\sqrt{N_{\text{total}}}} \tag{A20}$$

Table A1 Performance Impact of Spatial Correlation

Correlation Level	N_{eff}	$\eta_{\text{correlation}}$	Error Reduction Loss
Uncorrelated ($\rho = 0$)	16.0	1.00	0%
Weak ($\rho = 0.2$)	14.1	0.94	6%
Moderate ($\rho = 0.5$)	10.7	0.82	18%
Strong ($\rho = 0.8$)	6.4	0.63	37%

A.6 Mitigation Strategies

A.6.1 Frequency Diversity

Using different frequency bands for corner radars on the same vehicle:

$$f_{i,j} = f_c + \Delta f \cdot j, \quad j = 1, 2, 3, 4 \tag{A21}$$

where $\Delta f = 1$ GHz provides decorrelation.

A.6.2 Polarization Diversity

Alternating vertical and horizontal polarizations for corner radars reduces correlation:

$$\rho_{\text{cross-pol}} = \rho_{\text{co-pol}} \times \text{XPD}^{-1} \tag{A22}$$

where XPD is the cross-polarization discrimination (typically 15-20 dB).

A.6.3 Temporal Diversity

Sequential measurements with time offset $\Delta t > \tau_c$ (coherence time) provide decorrelation:

$$\tau_c = \frac{1}{2\pi f_D} = \frac{\lambda}{2v_{\text{rel}}} \tag{A23}$$

For $v_{\text{rel}} = 100$ m/s and $\lambda = 5$ mm : $\tau_c = 25$ μs.

To conclude this, the comprehensive analysis demonstrates that:

1. **Corner radar spacing**: The 2-4 m separation between corner radars ensures minimal spatial correlation for 60 GHz systems.
2. **Multipath effects**: Ground reflection introduces small range biases (~ 0.07 m) that are negligible compared to measurement noise.
3. **Robustness**: Even with moderate correlation ($\rho = 0.5$), the corner radar system maintains 82% of its uncorrelated performance.
4. **Practical validity**: The independent noise assumption used in the main CRLB analysis is well-justified for the proposed corner radar geometry.

The theoretical framework thus remains valid, with spatial correlation effects providing less than 20% performance degradation in realistic scenarios, confirming the robustness of the multi-vehicle FMCW radar approach for active protection systems.

Appendix B Sensor Synchronization Requirements and Error Analysis

The multi-vehicle FMCW radar system requires precise time synchronization between distributed sensors to enable coherent data fusion. This appendix analyzes synchronization requirements and proposes practical implementation approaches.

B.1 Synchronization Error Model

Consider timing errors Δt_i at vehicle i relative to a common reference. The measured range becomes:

$$\tilde{R}_i(t) = R_i(t + \Delta t_i) \approx R_i(t) + \dot{R}_i(t)\Delta t_i \tag{B24}$$

For projectile tracking, the range rate $\dot{R}_i(t)$ can reach ±300 m/s. Therefore, timing errors directly translate to range measurement errors:

$$\delta R_i = \dot{R}_i \cdot \Delta t_i \tag{B25}$$

B.2 Error Propagation to Parameter Estimation

Using the CRLB framework, synchronization errors increase the measurement covariance matrix:

$$\mathbf{\Sigma}_{sync} = \mathbf{\Sigma}_{thermal} + \text{diag}(\sigma^2_{sync}, 0) \tag{B26}$$

where $\sigma^2_{sync} = (\dot{R}_i \cdot \sigma_{\Delta t})^2$ and $\sigma_{\Delta t}$ is the RMS timing error.

The degradation in pass range estimation accuracy is:

$$\frac{\text{CRLB}_{sync}}{\text{CRLB}_{ideal}} = \frac{\sigma^2_{thermal} + \sigma^2_{sync}}{\sigma^2_{thermal}} = 1 + \frac{\sigma^2_{sync}}{\sigma^2_{thermal}} \tag{B27}$$

B.3 Synchronization Requirements

To limit performance degradation to 10%, we require:

$$\frac{\sigma^2_{sync}}{\sigma^2_{thermal}} < 0.1 \tag{B28}$$

For our 60 GHz FMCW system with $\sigma_{thermal} \approx 0.05$ m (from Section **??**):

$$\sigma_{sync} < 0.1 \cdot \sigma_{thermal} = 0.005 \text{ m} \tag{B29}$$

$$\sigma_{\Delta t} < \frac{0.005}{300} = 17 \text{ ns} \tag{B30}$$

Synchronization Requirement: $\sigma_{\Delta t} < 17$ ns RMS

B.4 Proposed Implementation Approaches

B.4.1 GPS-Disciplined Oscillators (GPSDO)

Approach: Each vehicle uses a GPS-disciplined crystal oscillator providing 10 MHz reference and 1 PPS timing [**?** **?**].

Performance:

- Absolute timing accuracy: ±100 ns (GPS limitation)
- Relative timing stability: ±1 ns over integration periods
- Holdover stability: < 10 ns drift over 30 seconds

Analysis: The 1 ns relative stability easily meets our 17 ns requirement for projectile tracking applications.

B.4.2 Inter-Vehicle Time Transfer

For improved relative timing, implement IEEE 1588 Precision Time Protocol (PTP) [**?** **?**] over vehicle-to-vehicle communication:

Two-Way Time Transfer Protocol:

1. Vehicle A transmits timestamp T_1 to Vehicle B
2. Vehicle B receives at time T_2, responds at T_3
3. Vehicle A receives response at T_4
4. Clock offset: $\theta = \frac{(T_2-T_1)-(T_4-T_3)}{2}$
5. Propagation delay: $\delta = \frac{(T_2-T_1)+(T_4-T_3)}{2}$

Achievable accuracy: < 1 ns over typical inter-vehicle distances (< 500 m).

B.5 Robustness Analysis

B.5.1 GPS Denial Scenarios

In GPS-denied environments, use crystal oscillator holdover:

- High-quality TCXOs: ±1 ppm stability
- Maximum tracking duration: $\frac{17 \text{ ns}}{1 \text{ ppm}} = 17$ seconds
- Sufficient for most engagement scenarios

B.5.2 Communication Link Failures

If inter-vehicle communication fails:

- Fall back to GPSDO-only synchronization
- Performance degrades by $\sqrt{2}$ factor but remains functional
- Individual vehicle tracking provides backup capability

B.6 Implementation Complexity Assessment

Recommendation: GPSDO-based synchronization provides optimal balance of performance, cost, and complexity for multi-vehicle active protection systems.

Table B2 Synchronization Implementation Options

Method	Accuracy	Cost	Complexity
GPS Standard	±100 ns	Low	Low
GPSDO	±1 ns	Medium	Medium
GPSDO + PTP	< 1 ns	Medium	High
Atomic Clock	±0.1 ns	Very High	Medium

B.7 Experimental Validation Approach

To validate synchronization performance:

1. **Benchtop Testing**: Use network analyzer to inject controlled timing offsets and measure tracking degradation
2. **Field Testing**: Deploy synchronized vehicles with common target and compare individual vs. fused estimates
3. **Metrics**: Measure actual timing jitter, range measurement consistency, and parameter estimation accuracy

The analysis demonstrates that multi-vehicle FMCW radar systems can achieve the required < 17 ns synchronization using commercially available GPS-disciplined oscillators. The proposed implementation is:

- **Feasible**: Standard GPSDO technology meets requirements
- **Robust**: Graceful degradation in GPS-denied scenarios
- **Cost-effective**: Moderate complexity and cost overhead
- **Scalable**: Approach extends to larger vehicle formations

This synchronization framework enables practical deployment of the theoretical performance improvements demonstrated in the main paper.
